# Myelin debris uptake by macrophages and microglia: Resolution of foam cells with a series of novel cyclodextrins

**DOI:** 10.1016/j.neurot.2026.e00943

**Published:** 2026-06-12

**Authors:** Emily C. Wuerch, Charbel S. Baaklini, Ping Zhang, Marlene T. Mørch, Austin Che, Charlotte D'Mello, Chang-Chun Ling, V. Wee Yong

**Affiliations:** a Hotchkiss Brain Institute and the Department of Clinical Neurosciences, University of Calgary, Calgary, Canada; b Department of Chemistry, University of Calgary, Calgary, Canada

**Keywords:** Phagocytosis, Macrophages, Cyclodextrins, Multiple sclerosis

## Abstract

Foamy, lipid-laden macrophages are found in multiple sclerosis (MS) lesions, resulting from excessive phagocytosis of myelin debris following demyelination. These lipid-laden macrophages exhibit an inflammatory phenotype, inhibit remyelination, and likely contribute to MS pathology, yet effective therapeutic strategies to target them are lacking. In this study, we sought to better characterize the temporal patterns of myelin debris phagocytosis by human and murine microglia/macrophages, and generate an *in vitro* model of foamy phagocytes. In addition, given their demonstrated ability to promote lipid efflux in models of other neurological diseases, we investigated cyclodextrins as potential therapeutic agents in MS. We hypothesized that cyclodextrins could lower the accumulation of lipids in foamy macrophages, potentially modulating their inflammatory phenotype. Several in house-synthesized cyclodextrins were evaluated. We found that prolonged application of myelin and inflammatory cytokines produced an inflammatory macrophage phenotype with an MS-like signature, as determined through bulk sequencing analysis. When particular cyclodextrins were applied to these foamy macrophages in culture, there was reduced accumulation of ingested and processed lipids, and altered expression of genes related to inflammatory pathways or wound healing. Our findings suggest that cyclodextrins may modulate the phenotype of inflammatory macrophages, typical of MS pathology, and hold therapeutic potential in MS, warranting further investigation.

## Introduction

Multiple sclerosis (MS) is an inflammatory disease of the central nervous system (CNS) characterized by neuroaxonal degeneration and demyelination, the latter giving rise to myelin debris in lesions. Phagocytosis of myelin debris is a desired outcome as it is associated with several benefits in MS. First, phagocytosis clears myelin debris from lesion sites, where, otherwise, its presence inhibits remyelination. Indeed, enhanced myelin debris clearance is associated with improved remyelination in animal models of MS [1,2]. Additionally, phagocytosis of myelin debris promotes an initial regulatory phenotype in macrophages, as myelin lipids activate signaling cascades through liver X receptor (LXR) and peroxisome proliferator-activated receptor-γ (PPARγ) receptors [[3], [4], [5]]. These regulatory macrophages exhibit reduced responsiveness to inflammatory stimuli [6], suppress autoreactive T cell proliferation [7], and modify their antigen presentation that results in T cells with reduced IFNγ production [8]. While phagocytosis of myelin debris by microglia and macrophages have desired outcomes, prolonged internalization of myelin fragments in aging or in pathology including MS can lead to a pro-inflammatory foamy phenotype of macrophages and microglia [3].

Foamy phagocytes were first identified in the context of atherosclerosis, when these lipid-laden, inflammatory macrophages were observed in atherosclerotic plaques. More recently, the concept of foam cells has been applied to CNS diseases, particularly MS where lesions contain foamy macrophages and microglia filled with myelin-derived lipids, giving them a distinctive foamy, vacuolated appearance and showing signs of dysregulated expression of lipid metabolism genes [9,10].

Foam cells are thought to be detrimental in MS. Compared to macrophages acutely exposed to myelin, foamy macrophages have elevated expression of inflammatory cytokines such as TNFα, IL-6 and IL-1β [9]. Additionally, foamy macrophages containing lipid droplets have reduced phagocytic capacity, impairing their ability to effectively contribute to the removal of toxic debris [11]. Intracellular cholesterol crystals within foam cells can also trigger inflammasome activation and lysosomal rupture [12], while persistent cholesterol trafficking to the endoplasmic reticulum (ER) can lead to ER stress and activation of the unfolded protein response [13,14]. Overall, foamy phagocytes contribute significantly to MS pathology, and represent a promising therapeutic target.

Cyclodextrins (CDs) are a class of therapeutics initially used as excipients to enhance the solubility and stability of drugs such as nicorette tablets and the antihistamine cetirizine [15]. CDs have also been explored as therapeutics for Niemann-Pick Disease Type C1 (NPC1), a genetic lysosomal storage disorder in which the absence of the NPC1 transporter prevents the transport of free cholesterol from lysosomes to downstream organelles such as the ER [16]. In NPC1 mouse models, treatment with 2-hydroxypropyl-β-cyclodextrin (HPβCD) reduces intracellular cholesterol accumulation, decreases neurodegeneration, and improves survival [[17], [18], [19]]. CDs have since been translated into clinical trials for NPC1 disease, where they promote cholesterol efflux from cells [20], reduce the severity of neurological symptoms [20], slow disease progression [21], and decrease markers of neurodegeneration including phosphorylated tau [22].

Altered lipid homeostasis has been implicated in several neurological and neurodegenerative disorders. Recently, CDs have been investigated for their potential therapeutic benefits in these conditions, including in models of Alzheimer's and Parkinson's disease, spinal cord injury, and stroke. In the ApoE4 transgenic model of Alzheimer's disease, HPβCD treatment reduces intracellular BODIPY accumulation within oligodendrocytes *in vitro*, and promotes myelination *in vivo,* accompanied by improvements in cognitive and neurological symptoms [23]. In an animal model of stroke, treatment with HPβCD reduces the presence of inflammatory infiltrates in the infarct area, decreases Oil Red O (ORO) lipid staining, enhances lipid metabolism, and mitigates neurodegeneration [24]. Similarly, in the MPTP model of Parkinson's disease, HPβCD treatment protects against neurodegeneration, increasing the survival of neurons in the midbrain [25]. Notably, while questions remain regarding the efficiency of cyclodextrins to cross the blood brain barrier, they are capable of ameliorating CNS pathologies when administered systemically [26].

While HPβCD has demonstrated therapeutic efficacy in people with NPC1 disease and in models of other neurological diseases, it could possibly be improved upon. While its primary mechanism involves promoting the clearance of lipids, particularly free cholesterol, from cells, its efficacy in clearing esterified lipids, such as those accumulating in foamy macrophages in MS, remains unclear. Moreover, hearing loss has been reported as a notable adverse effect among patients taking HPβCD [20], highlighting the need for improved formulations that maintain therapeutic benefits while minimizing side effects.

Throughout this study, we aimed to better characterize myelin phagocytosis by human and mouse microglia and macrophages, and to generate inflammatory, foamy macrophages resembling those found in MS lesions. We then sought to determine whether CDs could facilitate the export of lipids from foamy macrophages, and to explore how this export could modulate foamy macrophage morphology. In addition, we aimed to identify novel CD compounds that could have greater therapeutic potential, compared to the previously-used HPβCD. This research lays the foundation for future work to modulate foamy macrophages to enhance repair in models of MS.

## Methods

### Mice

Female 8-10-week-old C57Bl/6 mice from Charles River were used for all macrophage cultures, while CD-1 mouse pups aged post-natal days 0–2 were used for microglia cultures. Mice were housed on a 12 h light/dark cycle with unlimited access to food and water. All experiments were conducted in accordance with the Canadian Council for Animal Care, and the guidelines of the animal facility of the University of Calgary.

### Generation of myelin fragments

C57Bl/6 mice were euthanized via cervical dislocation, and brains were extracted and kept in phosphate-buffered saline (PBS) on ice. Brains were added to 0.32 M sucrose and homogenized, generating a 10% brain suspension. Brain homogenate was layered on top of 0.85 M sucrose, creating a sucrose gradient that was spun for 40 min at 20,000 g. Myelin was collected from the interface layer and spun twice in distilled water for 15 min at 20,000 g. Bicinchoninic acid (BCA) assay was performed to determine protein concentration of myelin, and myelin was diluted to 300 μg/ml in Live Cell Imaging Solution (Invitrogen).

### Macrophage culture

C57Bl/6 mice were euthanized via cervical dislocation, allowing for extraction of their tibias and femurs. The ends of each bone were removed, and a 25-gauge needle filled with media was used to flush bone marrow into a tube. Bone marrow was spun at 300 g for 8 min, and cells were counted and plated in 100 mm dishes at a density of 1 × 10^6^ cells/ml in 10 ml volume. These bone marrow-derived macrophages (BMDMs) were grown in DMEM (Gibco; 11960–069) with 1% glutamax, 1% pen/strep, 10% FBS and 10% L929 media and cultured at 37 °C with 8.5% CO_2_. Half of the media was changed on d5, and all of the media was changed on d7. On d8, media was removed, ice-cold PBS was applied, and cells were scraped into a tube. Cells were spun at 300 g for 8 min and counted. Macrophages were plated in 96 well, flat-bottom black/clear plates at a density of 30,000/well in 100 μl volume, and moved to 5% CO_2_. For experiments where ORO staining was performed, 5 mm glass coverslips (Bellco; 72195) were added to wells prior to seeding. Macrophages were exposed to myelin two days post-seeding.

### Murine microglia culture

T75 culture flasks were coated with 10 μg/ml poly-d-lysine (Thermofisher) at 37 °C for at least 30 min. Culture medium, consisting of DMEM (Gibco; 11960–044) with 10% FBS, 1% glutamax, 1% pen/strep and 1% non-essential amino acids, was prepared. CD-1 mouse pups aged between postnatal days 0–2 were decapitated, and their brains were removed and put in ice-cold HBSS (Gibco; 14175–079). Under the dissection microscope, meninges and cerebellum were removed, and cortices were transferred to HBSS on ice. Tissue was centrifuged at 150 g for 1 min, and manually homogenized in culture media, generating a single cell suspension. The suspension was centrifuged at 400 g for 5 min, and the pellet was resuspended in culture media. Cells were seeded at a density of 1 mouse pup per T75 flask in 10 ml culture media, and incubated at 37 °C with 5% CO_2_. Cells underwent a full media change on d5, and a half media change on d10. When the microglia were ready (typically between days 10–20), floating cells were washed from the flask and harvested, and then centrifuged for 5 min at 400 g. Microglia were resuspended in 1 ml of culture media, counted, and plated in 96-well, flat-bottom black/clear plates at a density of 30,000/well in 100 μl volume. For experiments assessing ORO staining, 5 mm glass coverslips (Bellco; 72195) were added to wells prior to seeding. After 1 h, media was changed to remove non-adherent cells including astrocytes and oligodendrocyte progenitor cells (OPCs). Murine microglia were exposed to myelin two days post-seeding.

### Human adult microglia culture

Complete MEM media containing 1% glutamax, 1% pen/strep, 1% non-essential amino acids, 1% sodium pyruvate, 0.1% dextrose and 10% FBS was prepared and warmed. Human adult brain tissues were obtained from surgical resections of epileptic foci. The sample was washed and trypsinized with 8 ml DNase (1 mg/ml) and 5 ml trypsin (2.5%) in a total volume of 50 ml PBS for 45 min at 37 °C. After digestion, 5 ml of FBS was added to inactivate trypsin, and the digested tissue was homogenized through a 130 μm filter and spun at 300 g for 10 min. Cells were resuspended in percoll (Sigma) and centrifuged at 22,500 g for 30 min. Following centrifugation, cells were collected from the middle interface and spun again at 300 g for 10 min. Cells were resuspended in complete MEM media and centrifuged twice at 300 g for 10 min. Lastly, cells were resuspended in complete MEM media, counted, and seeded at 2 million cells/ml in 5 ml complete MEM media in uncoated T25 flasks. Media was changed 4 h after seeding and again 2–3 d later. Microglia were ready for harvest between days 5–7. To harvest, a trypsin solution consisting of 3 ml versine, 2 ml DNase (1 mg/ml) and 0.5 ml Trypsin (2.5%) was generated, and 2 ml was added to each T25 flask. Flasks were incubated with trypsin solution for 2–3 min at 37 °C, 5% CO_2_. After incubation, cells were washed with PBS, and the cell suspension was added to complete MEM to inactivate the trypsin. Cells were centrifuged at 300 g for 10 min, counted, and plated in 96 well, flat-bottom black/clear plates at a density of 25,000/well in 100 μl volume. For experiments where ORO staining was performed, 5 mm glass coverslips (Bellco; 72195) were added to wells prior to seeding. Human microglia were exposed to myelin two days post-seeding into 96-well plates.

### Phagocytosis assay

To assess phagocytosis of myelin debris, full media was removed, and 50 μl of starved media (1% FBS) was added. After 1 h of serum starvation, 50 μl of starved media containing myelin (60 μg/ml) was added on top, at twice the desired concentration. Control cells received 50 μl of starved media, without myelin. After timepoints between 2 and 72 h, media was removed and replaced with 100 μl of 4% PFA for 15 min. Fixed cells were washed with PBS and stored at 4 °C until used for immunocytochemistry.

### Generation of foamy macrophages

To generate a foamy macrophage phenotype, macrophages in wells of 96-well plate were treated with myelin and/or inflammatory cytokines IL-1β and IFNγ for 6 h or 3 d. Full 10% FBS-containing media was removed and replaced with 50 μl of starved media (1% FBS). After 1 h of serum starvation, 50 μl of starved media containing myelin (60 μg/ml), with or without IL-1β (20 ng/ml) and IFNγ (20 ng/ml) was added on top, at twice the desired concentration. After 6 h or 3 d of incubation, media was removed and replaced with 100 μl of 4% PFA for 15 min. Fixed cells were washed with PBS and stored at 4 °C until used for immunocytochemistry.

### Cyclodextrins (CDs)

HPβCD was purchased from Sigma-Aldrich (H107). Other CD derivatives were generated in-house according to previously published procedures: AC3119, AC3147 and AC380 according to Che et al. [27]; PZ7061, PZ7084, PZ7086 and PZ7095 according to Ling et al. [28]; PZ8059, PZ8061, PZ9078 and PZ9080 according to Ling et al. [29] CDs were diluted in sterile water to generate a stock solution of 250 mM and stored at 4 °C until use. For acute experiments, microglia/macrophages were subjected to serum starvation for 1 h with 50 μl of 1% FBS macrophage (starved) media. After serum starvation, 50 μl of starved media containing myelin (60 μg/ml) was added on top. After 6 h, myelin was removed, and CDs were added in starved media at a concentration of 1 or 5 mM. After 24 h, media was removed and cells were fixed with 4% PFA. Fixed cells were stored at 4 °C until use in immunocytochemistry. For chronic experiments, macrophages were subjected to serum starvation for 1 h with 50 μl of 1% FBS macrophage media. After serum starvation, 50 μl of starved media containing myelin (60μg/ml), IL-1β (20 ng/ml) and IFNγ (20 ng/ml) was added on top. After 3 days, when cells had adopted a foamy phenotype, CDs were added in starved media at 1 or 5 mM concentration. After 48 h treatment, media was removed, and cells were fixed with 4% PFA. Fixed cells were stored at 4 °C until use in immunocytochemistry.

### Cholesterol detection in cell culture supernatant

The Amplex™ Red Cholesterol Assay Kit (Invitrogen; A12216) was used for evaluation of cholesterol content in supernatant from cyclodextrin-treated macrophages. The Amplex reagent was added to supernatant according to package instructions, incubated for 30 min at 37 °C, and immediately read with a fluorescent plate reader. To investigate whether cyclodextrins were sequestering cholesterol, cyclodextrins (5 mM) were added directly to the cholesterol standard, and incubated at room temperature for 2 h. After incubation, the Amplex reagent was added to supernatant according to package instructions, incubated for 30 min at 37 °C, and immediately read with a fluorescent plate reader.

Levels of cholesterol in culture medium were also assessed using mass spectrometry. Ten μL of cell-conditioned medium was mixed with 100 μL of internal standard solution, followed by another 90 μL of 90% MeOH: H_2_O (*v/v*). The solution was centrifuged at 14,000 g for 10 min and 180 μL of supernatant was then submitted for analysis using an Agilent 1290 binary liquid chromatography connected with an AB SCIEX QTRAP 5500 mass spectrometer equipped with electrospray ionization source. Liquid chromatography separation was performed on an Agilent ZORBAX Eclipse plus C18 column (100 × 2.1 mm, 1.8 μm particle size) at 40 °C. Mobile phase A was 50% MeOH/H_2_O (*v/v*, 10 mM NH_4_OAc, 0.1% formic acid) and mobile phase B was 100% MeOH (10 mM NH_4_OAc, 0.1% FA). The 10 min gradient was 90% B (0–0.1 min), 98–100% B (0.1–4 min) and 100% B (4–10 min). Flow rate was 200 μL/min. Injection volume was 5 μL. Between each injection, the column was equilibrated at 90% mobile phase B for 4 min.

### Immunocytochemistry

Fixed cells were permeabilized with 0.2% Triton X for 10 min and blocked with Odyssey blocking buffer (LI-COR) at room temperature for 1 h. After blocking, cells were incubated with the following primary antibodies overnight at 4 °C: LAMP1 (1:400; sc-19992), myelin basic protein (MBP; 1:500; PA1-10008), and Plin2 (1:500; GP40). The next day, cells were washed and incubated with the following secondary antibodies or fluorescently-conjugated dyes for 1 h at room temperature: Alexa Fluor 488 donkey anti-chicken (1:400; 703-545-155), Alexa Fluor 594 donkey anti-rat (1:400; 712-545-150), Alexa Fluor 647 goat anti-guinea pig (1:400, A-21450), BODIPY (1:1000; D3922), DAPI (1:1000; D1306), Phalloidin 546 (1:600; A22283), Phalloidin 594 (1:400; A12381), Phalloidin 647 (1:400; A22287). After incubation, cells were washed and stored in PBS at 4 °C until imaging.

### Filipin staining

To visualize intracellular free cholesterol, cells were stained with filipin (Sigma). Fixed cells were permeabilized with 0.2% Triton X for 10 min at room temperature. Following permeabilization, Filipin (1:20; SAE0087) and DRAQ7 (1:100; ab109202) were added to DMSO (1:4) and PBS for 2 h and incubated at room temperature. Following incubation, cells were washed with PBS and immediately imaged to prevent signal decay.

### Oil red O (ORO) staining

ORO staining was used to visualize esterified cholesterol. Fixed cells were exposed to 60% isopropyl alcohol for 10 min. Isopropyl alcohol was removed and ORO solution consisting of 0.3% ORO (Sigma; O0625-25G) in 60% isopropyl alcohol was added and incubated for 10 min. Cells were washed twice with water and hematoxylin was added for 10 s. Cells were again washed twice with water, and coverslips were flipped onto glass slides and mounted in Aquatex mounting media (Sigma). Cells stained with ORO were imaged immediately to prevent loss of signal.

### Microscopy

Cells stained with BODIPY, Plin2, MBP, or Filipin were imaged at 10X magnification with the ImageXpress Micro XLS High-Content Analysis System. Consistent exposure parameters were used for all groups within the same experiment. Twelve images were taken per well for quantitative analysis. Cells stained with ORO were imaged at 20X magnification with the Olympus VS110 Virtual Slide Scanner. One image containing the entire coverslip was taken per well. Representative images were taken at 25X with the Leica TCS SP8 Confocal Microscope, zoom 4, unless otherwise indicated.

### Image analysis

Multiwavelength cell scoring analysis in the MetaXpress High-Content Image Acquisition and Analysis Software (Molecular Devices) was performed to quantify the number of DAPI + cells, the percentage of DAPI + microglia and macrophages that were also positive for lipid markers, and to quantify mean cell (phalloidin+) and lysosomal (LAMP1+) area. Cell viability following cyclodextrin treatment was evaluated as the number of DAPI + cells. Consistent analysis parameters were used across all images within the same experiment. Cells stained with ORO were analysed using QuPath. Classifiers for ORO+ and ORO- cells were generated and the program was trained to recognize ORO + cells. Trained classifiers were applied to the ROIs containing ORO-stained cells. Classifier parameters were kept consistent across all images from the same experiment, and the percentage of hematoxylin + cells that were also ORO+ was recorded.

### Cytokine assay

Supernatant was collected from cells at timepoints of interest and frozen at −80 °C until use. Media was used in either a Mouse Cytokine/Chemokine 32-Plex Discovery Assay® Array, or Mouse Cytokine/Chemokine 36-Plex Discovery Assay® Array (Eve Technologies), which were performed according to manufacturer instructions.

### RNA isolation for bulk sequencing

BMDMs were generated as described above, and cells were plated in 6-well plates at a density of 500,000/well in 2 ml full macrophage medium (4 wells per condition, serving as technical replicates). After 48 h, media was removed and replaced with 1 ml starved (1% FBS) macrophage medium. One hour later, 1 ml of starved media containing myelin (60 μg/ml), IL-1β (20 ng/ml) and IFNγ (20 ng/ml) was added on top. Following three days of stimulation to generate a foamy macrophage phenotype, stimulated media was removed, and replaced with 1 ml of I) starved macrophage media, II) stimulated starved macrophage media containing myelin, IL-1β and IFNγ, III) stimulated starved macrophage media with PZ8059 at a concentration of 1 mM or IV) stimulated starved macrophage media with HPβCD at 1 mM. After 6 h treatment with CDs, media was removed, wells were washed with PBS, and 1 ml of Trizol was added per well. After 3 min, Trizol and cell lysate were removed and frozen at −80 °C. The next day, samples were thawed and aspirated with 30 G needle. 200 μl of chloroform was added to each tube, and tubes were well mixed. Tubes were spun at 9000 g for 20 min. Aqueous phase containing RNA was collected and precipitated with 70% ethanol. RNA was collected with RNeasy Mini Kit Columns (Cat: 74104, Qiagen), and the NanoDrop Spectrophotometer (ThermoFisher) was used to determine RNA concentration and purity.

### Bulk RNA sequencing analysis

Total RNA from each sample was used to prepare mRNA seq libraries with poly A capture using the NEBNext ULTRA EXPRESS RNA Library Prep KiNEB with poly-A enrichment (New England Biolabs). Libraries were sequenced using a NextSeq 2000 P3 XLEAP flowcell (Illumina) at a depth of 33 M reads/library. FastQC (v0.11.5) was used to determine the quality of sequencing reads and cutAdapt (v1.12) [30] was used for quality trimming (q20). Kallisto (v0.46.2) [31] was used for quantifying abundances of transcripts. Kallisto index was built with reference transcriptome GRCh39. The R package tximport [32] was used to summarize transcript level abundances from kallisto to gene level. The R package DeSeq2 [33] was used for differential expression analysis (DEG). Principal component analysis (PCA) plots were generated using rlog-transformed data. To generate lists of top differentially expressed genes (DEGs) (Supplementary Tables 1–4), all DEGs with adjusted p value > 0.05 were removed. Significant DEGs were then filtered for maximum log2foldchange. For the formation of volcano plots, data underwent LFC shrinkage using ashr method [34]. Volcanos were generated using EnhancedVolcano in R, and differentially expressed genes were plotted based on cut-offs of padj<0.05, and log2 fold change ± 0.5 [35]. Heatmaps were generated with pheatmap in R, using rlog-transformed data [36].

DEGs were analysed with the use of QIAGEN IPA (QIAGEN Inc., https://digitalinsights.qiagen.com/IPA) [37]. When conducting Core Expression Analyses, observations of interest were log2 fold change (expression log ratio) and adjusted p-value (expression p value). Expression analyses were performed based on log2 fold change, with an adjusted p-value threshold of 0.05. For control vs stimulated, a log2 fold change threshold of ± 1 was applied. For other comparisons, a threshold of ± 0.5 was used.

### Statistical analysis

Analysis and graphs were generated using GraphPad Prism 9 (LaJolla, CA). Two-tailed, unpaired t-tests were used to analyse the differences between two groups. One-way ANOVAs with Tukey's post-hoc test were used to analyse the differences between three or more groups, with one independent variable. Two-way ANOVAs with Fisher's least significance difference test were used to analyse the differences between three or more groups, with two independent variables. Data is shown as mean±SD. The p-value is represented by the number of asterisks: ∗*p* < 0.05, ∗∗*p* < 0.01, ∗∗∗*p* < 0.001, ∗∗∗∗*p <* 0.0001.

## Results

### Myelin phagocytosis leads to an initial increase in intracellular myelin and free cholesterol, followed by an accumulation of lipid droplets

**Fig. 1:**
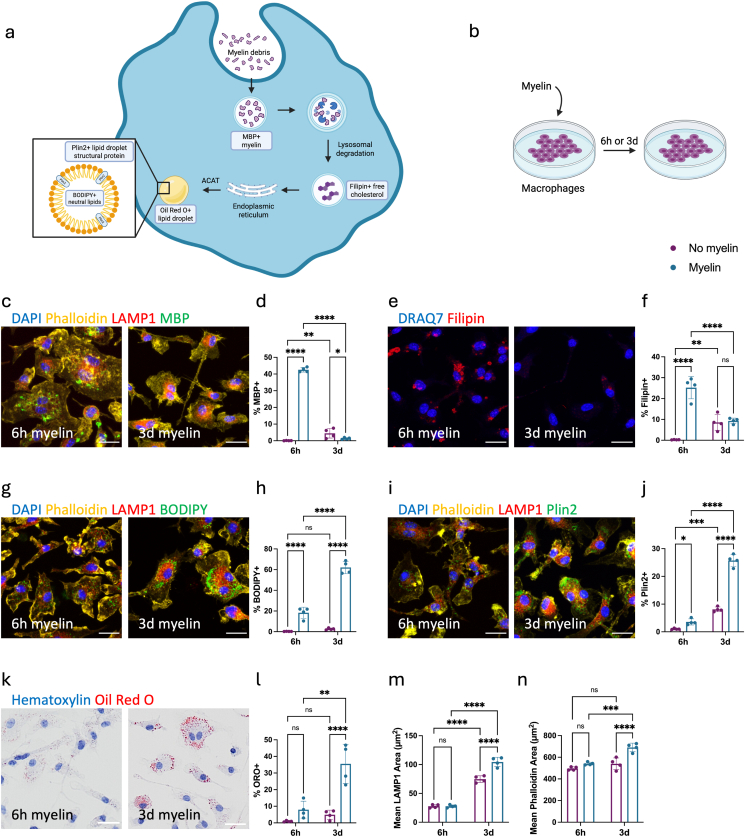
**Characterizing uptake of myelin debris by murine macrophages at acute and longer timepoints. a)** Schematic of lipid markers detected following myelin phagocytosis. Ingested myelin is degraded within lysosomes, generating free cholesterol, which is then transported to downstream organelles including the endoplasmic reticulum. In the endoplasmic reticulum, lipids are esterified into lipid droplets. **b)** Schematic of macrophage phagocytosis assay. **c, e, g, i, k)** Representative images of macrophages exposed to myelin for 6 h or 3 d, stained with MBP **(c)**, Filipin **(e)**, BODIPY **(g)**, Plin2 **(i)**, or ORO **(k)**. Representative images of ORO staining were captured on the Olympus VS110 Virtual Slide Scanner at 20X magnification. **d, f, h, j, l)** Quantification of the percentage of cells positive for MBP **(d)**, Filipin **(f)**, BODIPY **(h)**, Plin2 **(j)**, or ORO **(k)**. **m)** Quantification of mean lysosomal (LAMP1+) area. **n)** Quantification of mean cell (Phalloidin+) area. N = 2 independent experiments; each experiment included 3–4 replicates. Mean±SD. ∗p < 0.05 ∗∗p < 0.01 ∗∗∗p < 0.001 ∗∗∗∗p < 0.0001. Two-way ANOVA with Fisher's LSD post-hoc test. Scale bar indicates 20 μm. Schematic created with BioRender.

After macrophages ingest myelin, the debris can be detected within lysosomes as MBP + signal. Within the lysosomes, myelin is broken down into its components, including cholesterol which cannot be further degraded and can be detected by filipin staining [9,38]. Cholesterol is transported from lysosomes to downstream organelles such as the ER [39]. Once in the ER, free cholesterol is esterified by the ACAT enzyme, forming lipid droplets that are ORO+ [40]. These lipid droplets consist of a monolayer of phospholipids and cholesterol, which also includes the structural protein Plin2, and a hydrophobic core of neutral lipids [41]. Neutral lipids such as cholesterol esters and triglycerides are detected by BODIPY staining (Fig. 1a).

Our objective was to temporally map the presence of various lipid markers in murine macrophages after exposure to myelin debris. We first conducted a preliminary experiment to determine key time points of interest. This experiment demonstrated that 6 h represents an acute phase, characterized by myelin staining and free cholesterol, while 3 d (72 h) serves as a representative chronic phase, characterized by the loss of free cholesterol and accumulation of esterified cholesterol (Fig. S1). In subsequent experiments, macrophages were exposed to myelin, and lipid markers were analysed at timepoints after 6 h or 3 d (Fig. 1b). In these macrophages, there was a sharp initial increase in MBP and Filipin staining, which declined by the three-day timepoint (Fig. 1c–f). Although BODIPY, Plin2 and ORO staining showed a modest increase at 6 h, a much larger signal was observed at the three-day timepoint, indicating a higher proportion of esterified lipids and lipid droplets at chronic timepoints compared to acute ones (Fig. 1g-l). We also identified a significant increase in lysosomal and cell area in myelin-exposed cells, when compared to unstimulated cells at 3 d (Fig. 1m and n). There was also an increase in lysosomal and cell area when comparing myelin-exposed cells at 6 h versus 3 d, indicating that prolonged myelin exposure induces a more ameboid phenotype (Fig. 1m and n). Overall, this work provides a temporal map of myelin degradation and lipid movement within macrophages following myelin exposure, while highlighting how myelin exposure impacts cell and lysosomal morphology.

### Characterization of lipid markers following acute and chronic myelin exposure in microglia

**Fig. 2:**
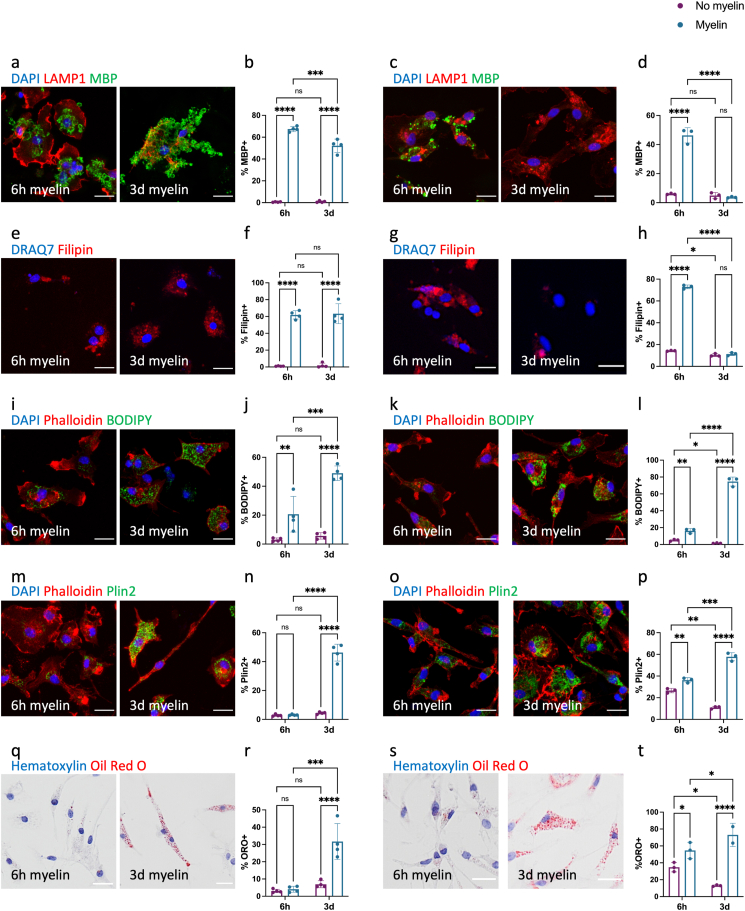
**Characterizing uptake of myelin debris by murine and human microglia at acute and chronic timepoints. a, e, i, m, q)** Representative images of murine microglia exposed to myelin for 6 h or 3 d, and stained with MBP **(a)**, Filipin **(e)**, BODIPY **(i)**, Plin2 **(m)**, or ORO **(q)**. **c, g, k, o, s)** Representative images of human microglia exposed to myelin for 6 h or 3 d, and stained with MBP **(c)**, Filipin **(g)**, BODIPY **(k)**, Plin2 **(o)**, or ORO **(s)**. Representative images of ORO staining were captured on the Olympus VS110 Virtual Slide Scanner at 20X magnification. **b, d, f, h, j)** Quantification of the percentage of cells that are positive for MBP **(b, d)**, Filipin **(f, h)**, BODIPY **(j, l)**, Plin2 **(m, p)**, or ORO **(r, t)**. N = 2 independent experiments; each experiment included 3–4 replicates for work on murine microglia. Experiments assessing adult human microglia involved only 1 specimen, 3–4 replicates per group. Mean ± SD. ∗p < 0.05 ∗∗p < 0.01 ∗∗∗p < 0.001 ∗∗∗∗p < 0.0001. Two-way ANOVA with Fisher's LSD post-hoc test. Scale bar indicates 20 μm.

Next, we aimed to further characterize the expression of lipid markers in murine and human microglia. In mouse microglia, we observed a similar large initial increase in MBP and Filipin staining; however, unlike macrophages, the signal remained elevated after 3 d of myelin exposure without a substantial decrease (Fig. 2a, b, e, f). Esterified cholesterol also increased after 3 d of myelin exposure, as indicated by a greater percentage of BODIPY+ (Fig. 2j), Plin2+ (Fig. 2n), and ORO + cells (Fig. 2r). Similarly, in human microglia, there was a large increase in MBP and free cholesterol, which decreased by the 3 d timepoint, mirroring what we observed in murine macrophages (Fig. 2c, d, g, h). We also saw a significant increase in esterified cholesterol at the chronic (3 d) timepoint, resembling the pattern we observed in murine macrophages and microglia (Fig. 2k, l, o, p, s, t). This work enabled us to build upon our work in murine macrophages and characterize myelin phagocytosis in human and murine microglia, and to compare and contrast how these cells differ in their patterns of myelin processing.

### Myelin alone is not sufficient to promote an inflammatory macrophage phenotype

We then aimed to generate foamy macrophages to better characterize the effects of prolonged myelin exposure on macrophage function and phenotype, serving as an *in vitro* model for subsequent experiments. To achieve this, we exposed macrophages to myelin for 6 h or 3 d, with unstimulated cells serving as controls. We analysed the cell culture supernatant for cytokines and chemokines to characterize the inflammatory nature of these cells (Fig. S2a). Surprisingly, myelin exposure alone did not significantly increase the expression of the majority of inflammatory molecules compared to the unstimulated controls (Fig. S2b-s). Indeed, there was minimal difference between the unstimulated and myelin-exposed groups, and also very little variation between the 6 h- and 3 d-myelin exposures, with the exception of the chemokine MIP (Fig. S2o and p). Consequently, we needed to modify our paradigm to generate foamy, inflammatory macrophages in culture.

### Myelin and cytokine exposure leads to increased lipid staining, enhanced ameboid morphology and an inflammatory phenotype

**Fig. 3:**
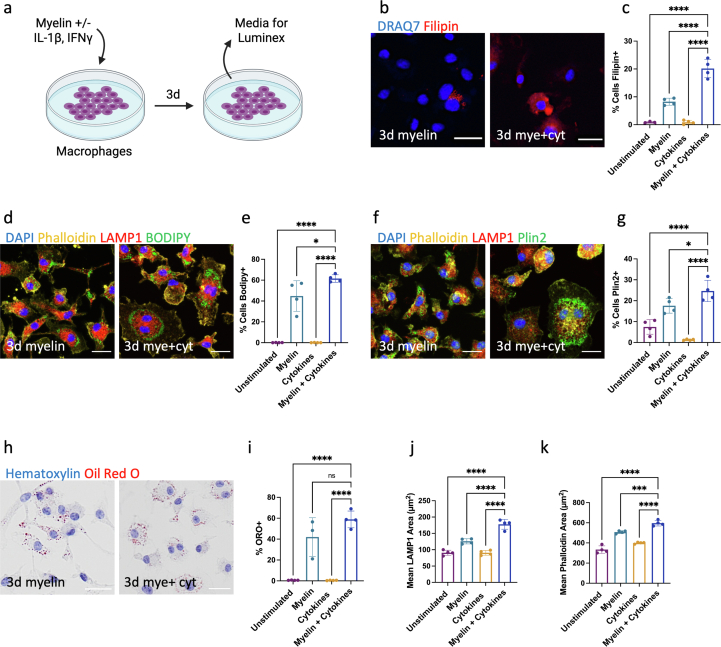
**Characterizing lipid markers and cell morphology in macrophages following exposure to myelin and/or cytokines. a)** Schematic of experimental design. **b, d, f, h)** Representative images of macrophages exposed to myelin, with or without cytokines, for three days and stained with Filipin **(b)**, BODIPY **(d)**, Plin2 **(f)**, or ORO **(g)**. Representative images of Filipin staining were captured on the ImageXpress Micro XLS High-Content Analysis System at 10X magnification. Representative images of ORO staining were captured on the Olympus VS110 Virtual Slide Scanner at 20X magnification. **c, e, g, i)** Quantification of the percentage of cells that are positive for Filipin **(c)**, BODIPY **(e)**, Plin2 **(g)**, or ORO **(i)**. **j)** Quantification of mean lysosomal (LAMP1+) area. **k)** Quantification of mean cell (Phalloidin+) area. N = 2 independent experiments; each experiment included 3–4 replicates. Mean±SD. ∗p < 0.05 ∗∗p < 0.01 ∗∗∗p < 0.001 ∗∗∗∗p < 0.0001. One-way ANOVA with Tukey's post-hoc test. Scale bar indicates 20 μm. Schematic created with BioRender.

We modified our approach by adding the inflammatory cytokines IL-1β and IFNγ, which are found in MS lesions [42,43]. In our revised paradigm, we exposed cells to myelin in the presence or absence of these cytokines, with unstimulated cells and cells treated with cytokines alone serving as control groups (Fig. 3a). We observed that cells exposed to both myelin and cytokines exhibited increased levels of intracellular free cholesterol, compared to those exposed to myelin alone (Fig. 3b and c). Additionally, there was an increase in the expression of lipid droplet markers BODIPY and Plin2, as well as a non-significant increase in the ORO signal, when comparing the myelin and cytokine group to myelin alone (Fig. 3d–i). We also found a significant increase in the cell (phalloidin+) area and lysosomal (LAMP1+) area following myelin and cytokine exposure (Fig. 3j and k). These findings indicate that macrophages maintain their capacity to phagocytose in the presence of cytokines, and are characterized by enhanced levels of intracellular free cholesterol, lipid droplets, and an ameboid morphology, compared to those exposed to myelin alone.

**Fig. 4:**
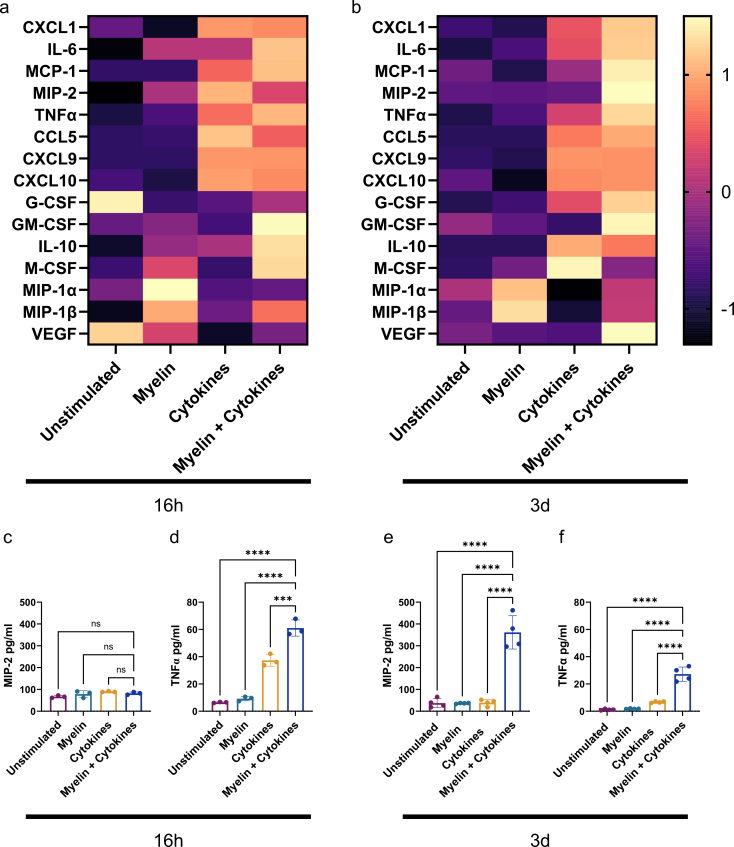
**Inflammatory cytokine and chemokine expression by macrophages following exposure to myelin and/or cytokines. (a, b)** Heatmap of cytokines and chemokines in the supernatant of macrophages exposed to myelin and/or cytokines at 16 h and 3 day time points, generated from replicate-averaged values followed by log transformation and z-score normalization. **(c**–**f)** Quantification of an example chemokine, MIP-2 **(c, e)**, and cytokine, TNFα **(d, f)**. Additional Luminex findings are presented in Supplementary Fig. 3. Mean±SD. ∗p < 0.05 ∗∗p < 0.01 ∗∗∗p < 0.001 ∗∗∗∗p < 0.0001. One-way ANOVA with Tukey's post-hoc test.

Next, we characterized the inflammatory nature of cells exposed to both myelin and cytokines by measuring cytokines and chemokines in the cell culture supernatant. We found that the combination of myelin and the inflammatory cytokines IL-1β and IFNγ had an additive effect on the expression of several cytokines such as IL-6 and TNFα, and chemokines including CXCL1, MCP-1 and CCL5, with elevations that were generally comparable between the 16 h and 3 day timepoints (Fig. S3 and Fig. 4). However, there were differences, such as MIP-2 that was increased by myelin and cytokines at 3 days but not 16 h (Fig. 3), or CXCL9 that was elevated by myelin and cytokines at 16 h but not at the later time point (Fig. S3). Together, these results demonstrate that exposure to myelin and cytokines generates an inflammatory macrophage phenotype at both early and late timepoints.

### Novel cyclodextrin formulations

**Fig. 5:**
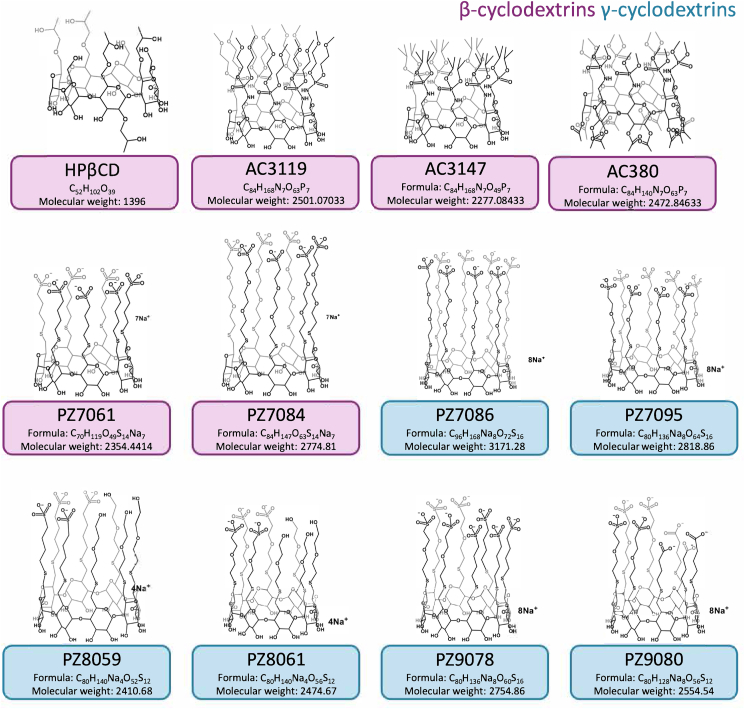
**Chemical structures of commercially available and novel cyclodextrins.** Chemical structures, formulas, and molecular weights of HPBCD and novel cyclodextrins. Purple indicates beta-cyclodextrins, while blue represents gamma-cyclodextrins. For PZ8059, PZ8061, PZ9078 and PZ9080, each of them consists of a mixture of approximately 1:1 ratio of the two types of indicated side chains, randomly substituted at the 6-positions of γCD.

Previous studies have predominantly assessed the therapeutic potential of HPβCD as this is commercially available. We generated eleven novel cyclodextrin formulations, to explore the potential for enhanced therapeutic properties (Fig. 5). While these formulations retained the characteristic barrel-shaped structure of CDs, they varied in their cavity sizes (AC3119, AC3147, AC380, PZ7061, PZ7084 are derived from the same β-CD as HPβCD, while PZ7086, PZ7095, PZ8059, PZ8061, PZ9078, PZ9080 are derived from γ-CD) and side chain compositions. For PZ8059, PZ8061, PZ9078 and PZ9080, each of them consists of a mixture of compounds randomly substituted by the two types of side chains in approximately 1:1 ratio at all the 6-positions of glucose units. The average molecular weights of the novel formulations ranged from 1884.33 g/mol to 3171.28 g/mol, and included both beta and gamma varieties. In contrast, the average molecular weight of HPβCD (degree of substitutions: 4–8) is 1396 g/ml.

### Cyclodextrins reduce intracellular lipid accumulation in microglia and macrophages

**Fig. 6:**
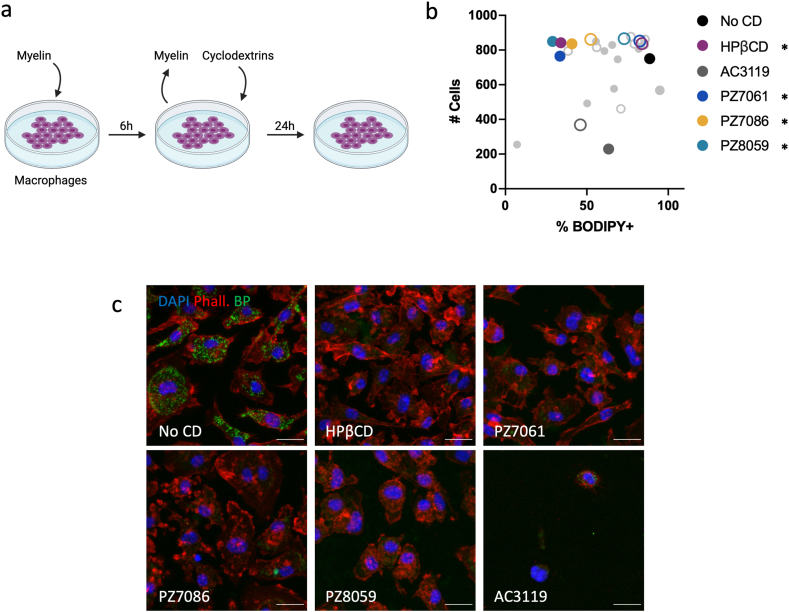
**Cyclodextrins reduce lipid accumulation within BMDMs. a)** Schematic of experimental design. **b)** Scatter plot representing the number of live macrophages versus the percentage of cells positive for lipid droplet marker BODIPY. Each dot represents one tested formulation, with closed circles representing 5 mM and open circles representing 1 mM concentration. For simplicity, only formulations of interest are depicted in color. Data is an average from two independent experiments. ∗Statistically significant from control (no CD) in degree of BODIPY staining. **c)** Representative images of untreated or cyclodextrin-treated macrophages stained with BODIPY. Scale bar indicates 20 μm. Images were taken from wells treated with 5 mM of cyclodextrins. Schematic created with BioRender.

We first evaluated the potential of HPβCD and novel CD formulations to reduce the content of lipids in myelin-exposed macrophages. Cells were incubated with myelin for 6 h, followed by medium change to remove excess myelin, and application of CDs for 24 h (Fig. 6a). Compared to untreated macrophages, several novel CD formulations, specifically PZ7061, PZ7086 and PZ8059, as well as the comparator HPβCD, significantly reduced BODIPY staining, without incurring cell death (Fig. 6b and c; S4a, b). For all formulations, a 5 mM concentration was more effective than 1 mM. While 88% of untreated macrophages were BODIPY+, this percentage decreased to 34% in HPβCD-treated, 33% in PZ7061-treated, 41% in PZ7086-treated, and 29% in PZ8059-treated macrophages. In contrast, other novel formulations, including AC3119, reduced BODIPY lipid droplets, but led to decreased cell numbers (Fig. 6b and c).

As MS lesions consist of microglia and peripheral macrophages, we next tested whether the most promising CD formulations in the above macrophage assays could reduce accumulated lipids from microglia as well. Using the same experimental paradigm as in macrophages, microglia were loaded with myelin for 6 h followed by fresh medium change, and then exposed to CDs for 24 h. In murine microglia, we observed that all tested formulations at a concentration of 5 mM led to a reduction in the presence of intracellular BODIPY + lipids without decreasing the number of cells (Fig. S5a and b). In human microglia, we again observed that all formulations reduced the percentage of BODIPY+ lipids at a concentration of 5 mM, when compared to untreated macrophages (Fig. S5c and d). No formulation at any concentration caused a significant decrease in cell number. Together, these results demonstrate that our novel CD formulations decrease lipid accumulation in myelin-exposed murine macrophages, as well as murine and human microglia, and show comparable efficacy to the previously published HPβCD.

### Cyclodextrins reduce lipid content within foamy macrophages

**Fig. 7:**
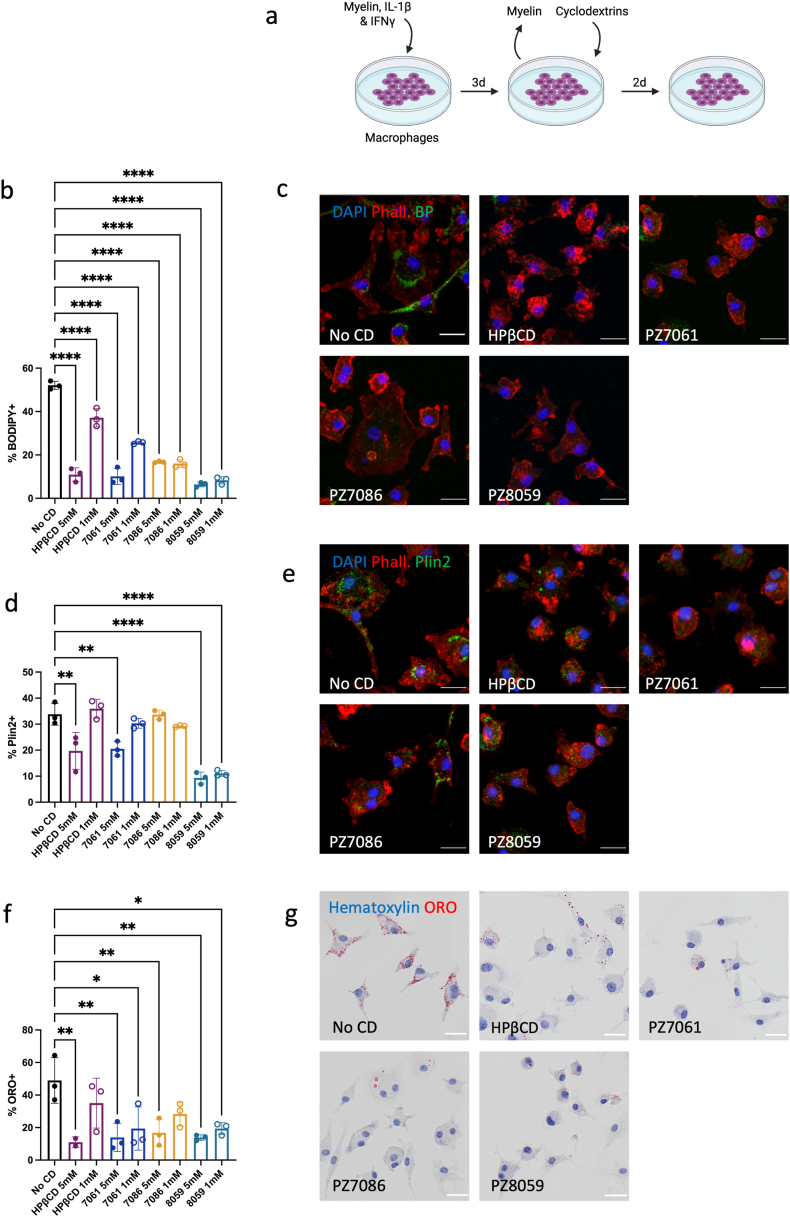
**Cyclodextrins decrease lipid content within foamy macrophages. a)** Schematic of experimental design. **b)** Quantification of the percentage of macrophages positive for lipid droplet marker BODIPY. **c)** Representative images of untreated or cyclodextrin-treated macrophages, stained with BODIPY (BP). **d)** Quantification of the percentage of macrophages positive for lipid droplet marker Plin2. **e)** Representative images of untreated or cyclodextrin-treated macrophages, stained with Plin2. **f)** Quantification of the percentage of macrophages positive for lipid droplet marker ORO. **g)** Representative images of untreated or cyclodextrin-treated macrophages, stained with ORO. Images of ORO were taken at 20X magnification on the Olympus VS110 Virtual Slide Scanner. All representative images were taken from wells treated with 5 mM of cyclodextrins. N = 2 independent experiments; each experiment included 3–4 replicates. Mean±SD. ∗p < 0.05 ∗∗p < 0.01 ∗∗∗∗p < 0.0001. One-way ANOVA with Tukey's post-hoc test. Scale bar indicates 20 μm. Schematic created with BioRender.

MS lesions are characterized by the presence of inflammatory, foamy microglia/macrophages, which contain esterified lipids [9]. We generated foamy macrophages as above through exposure to myelin and inflammatory cytokines, and cells were then treated with CDs for 48 h (Fig. 7a). Lipid content in these foamy macrophages was assessed using three markers for esterified lipids: BODIPY, Plin2 and ORO. All formulations, at concentrations of both 5 mM and 1 mM, reduced the percentage of BODIPY + cells compared to untreated controls (Fig. 7b and c). We next examined Plin2, a marker for the structural protein in lipid droplets. Compared to untreated macrophages, treatment with HPβCD, PZ7061, and PZ8059 at 5 mM reduced Plin2 staining. PZ8059 was the only formulation to reduce Plin2 at 1 mM (Fig. 7d and e). Notably, PZ7086 did not lower Plin2 staining at either concentration. Finally, we assessed ORO, another marker of esterified lipids. All formulations decreased ORO staining at 5 mM, while only PZ7061 and PZ8059 reduced ORO profiles at 1 mM (Fig. 7f and g). These findings build on our previous experiments and demonstrate that CDs can resolve accumulated lipids in foamy macrophages. Importantly, this also allowed us to better identify our lead compound, PZ8059, which out-performed HPβCD in lowering the density of detectable lipids in foamy macrophages.

### CDs sequester cholesterol in cell culture supernatant

To further investigate lipid clearance from CD-treated cells, we used the Amplex Cholesterol Assay to measure cholesterol levels in cell culture supernatants. We measured cholesterol content in the supernatant of untreated, PZ8059-treated, or HPβCD-treated foamy macrophages. Surprisingly, we observed a significant reduction in cholesterol levels in the supernatant from CD-treated macrophages, compared to untreated controls (Fig. S6a). We hypothesized that CDs were sequestering cholesterol in cell culture supernatant, preventing its detection by the Amplex assay.

To test this hypothesis, we set up a standard curve for the Amplex assay, and added CDs to some of the standard wells. After incubating for 2 h, we performed the assay as before. The standard wells not exposed to CDs showed the expected cholesterol concentrations of 8 μg/ml and 4 μg/ml. In contrast, the standard wells that were exposed to CDs for 2 h showed a significant reduction in the level of detected cholesterol, when added to both 8 and 4 μg/ml concentrations (Fig. S6b). Notably, PZ8059 caused the sharpest decline in cholesterol detection, which was significantly lower than HPβCD at both concentrations. These results demonstrate that CDs sequester cholesterol in cell culture supernatant, preventing its detection by the Amplex assay. Thus, if there was elevated lipid efflux from CD-treated cells, this was masked by the CD in the culture medium.

Next, we used liquid chromatography-mass spectrometry to measure cholesterol content in the supernatant of untreated and PZ8059-treated foamy macrophages, as this provided a method that detects cholesterol based on its mass. As with the Amplex Red results, cholesterol concentration was lower in all 3 samples of PZ8059-treated compared to control cells (Fig. S6c), although the paired *t*-test result did not show a statistical difference (p = 0.16). As the conditions of the mass spectrometry were not designed to measure cholesterol complexed to CDs, altogether, we were unable to resolve whether there was efflux of cholesterol from macrophages treated with CDs.

### Cyclodextrin treatment alters gene expression in foamy macrophages

Next, we conducted bulk RNA sequencing to further explore the mechanisms of CDs. Foamy macrophages were cultured as described above, and stimulation (myelin and cytokines) was removed to generate control cells. Stimulated cells were continuously exposed to myelin and inflammatory cytokines for another 6 h, while CD-treated cells during this period were exposed to both myelin and cytokines in the presence of CDs (HPβCD or PZ8059).

**Fig. 8:**
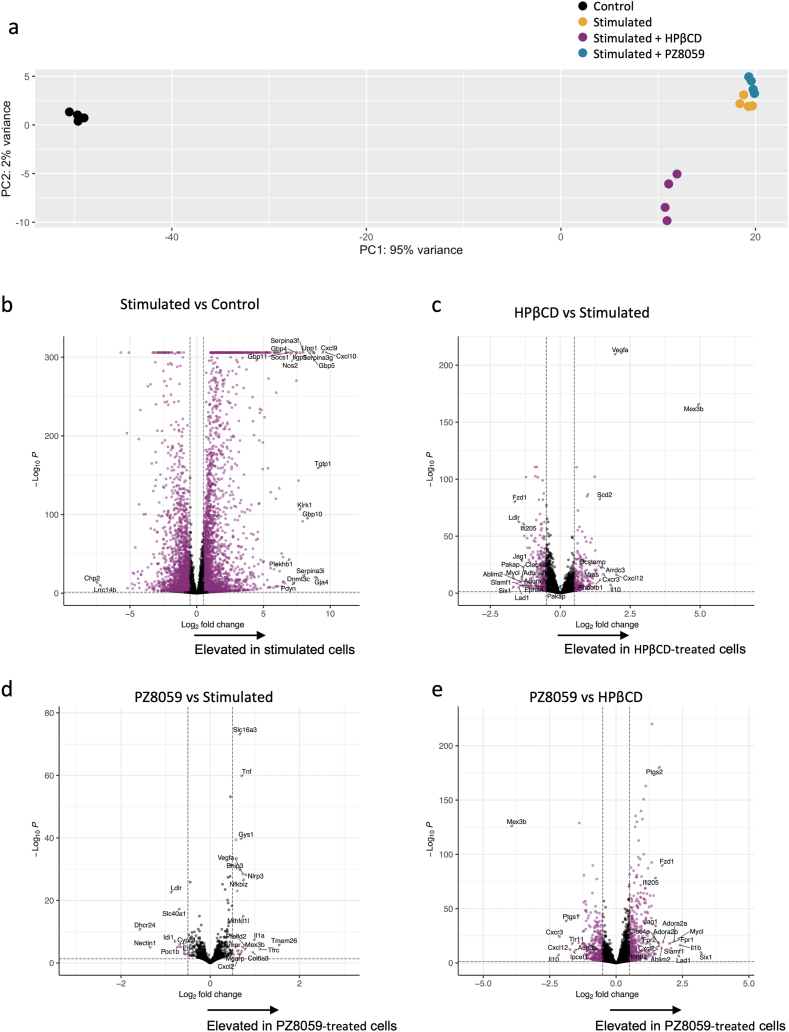
**Differentially expressed genes in foamy and cyclodextrin-treated macrophages**. **a)** PCA plot generated from rlog-transformed data. **b-e)** Volcano plots of comparisons of interest. Colored dots represent differentially expressed genes, using cut-off values of padj<0.05 and log2 fold change ± 0.5. Top 25 differentially expressed genes from each comparison are labeled. N = 4 technical replicates per group.

PCA analysis revealed minimal variation within each group but a clear distinction between control cells and all other conditions. Minimal differences were observed between stimulated and PZ8059-treated cells, while more pronounced differences were found between stimulated and HPβCD-treated cells (Fig. 8a). This trend was also reflected in the volcano plots, which showed the largest number of DEGs between stimulated and control cells, and the smallest number of DEGs between stimulated and PZ8059-treated cells (Fig. 8b–e). Comparing stimulated cells to control cells, key DEGs of interest that were upregulated in stimulated cells include *Cxcl9*, *Cxcl10*, and *Nos2,* which are all involved in inflammation (Fig. 8b).

In HPβCD-treated cells compared to stimulated cells, upregulated genes of interest include *Vegfa*, as well as cytokines/chemokines *Il10* and *Cxcl12*, and the inflammatory gene *Mex3b* (Fig. 8c). Conversely, genes such as *Ldlr* (low-density lipoprotein receptor), *Jag1* (Jagged1) and *Ifi205* (interferon-activated gene 205) were downregulated in HPβCD-treated cells, versus stimulated (Fig. 8c). In PZ8059-treated cells, inflammatory genes such as *Tnf*, *Nlrp3* and *Il1a* were upregulated compared to stimulated, while genes associated with lipid metabolism and cholesterol synthesis, including *Ldlr* and *Dhcr24* were downregulated (Fig. 8d). Direct comparisons between the two CD formulations revealed that cytokines/chemokines such as *Il10*, *Cxcr3* and *Cxcl12*, as well as the inflammatory gene *Mex3b* were downregulated in PZ8059-treated cells. Conversely, *Il1b*, *Jag1* and *Ifi205* were upregulated (Fig. 8e). Lists of top 50 DEGs for each comparison of interest are displayed in Supplementary Tables 1–4.

To further analyse transcriptomic changes, we used a heat map displaying the top 50 most variable genes across all groups. Hierarchical clustering confirmed that samples within the same group were most similar, aligning with the PCA results (Fig. S7). The most significant differences were observed between control cells and all other groups. Genes highly expressed in all stimulated cells but not in control cells included *Tfe3*, *Ins2* (Insulin 2), and *Apoh* (apolipoprotein H), which are linked to lipid metabolism, as well as *Slfn2*, which promotes inflammation (Fig. S7).

### Pathway analysis

**Fig. 9:**
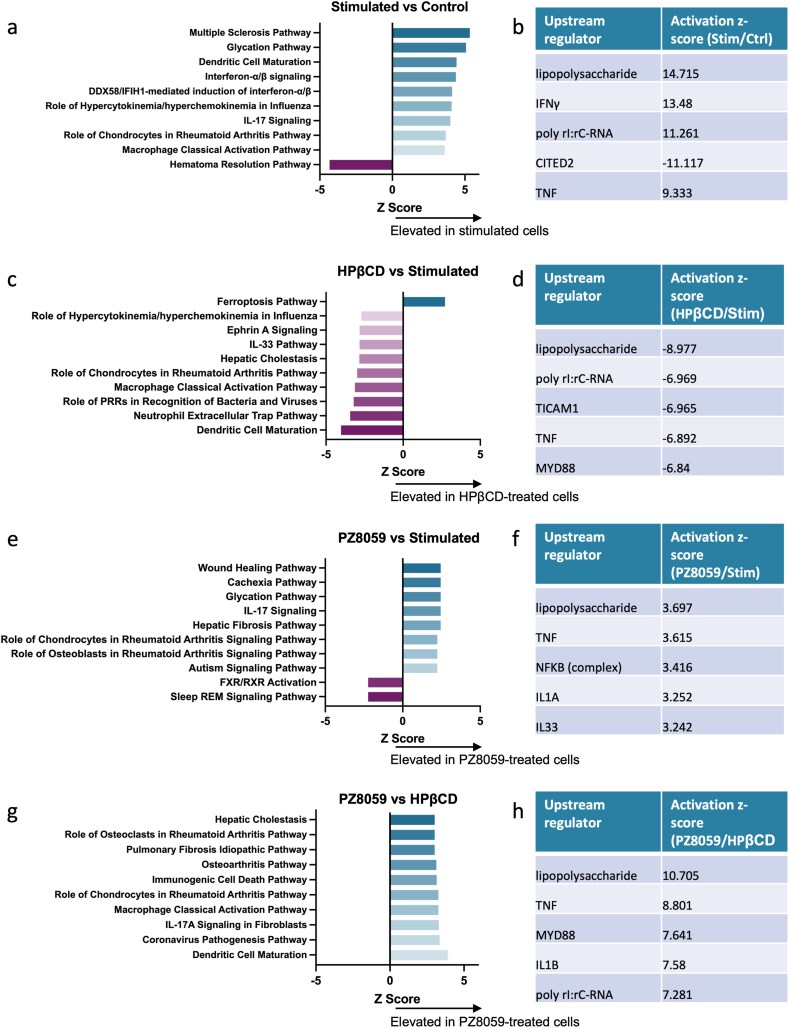
**Ingenuity pathway analysis in foamy and cyclodextrin-treated macrophages. a, c, e, g)** Top 10 altered pathways for each comparison of interest, as determined by z score. **b, d, f, h)** Top 5 upstream regulators for each comparison of interest, as determined by *z* score.

DEGs were also analysed using IPA (Qiagen) to assess pathway changes. MS and classical macrophage activation pathways were upregulated in stimulated (foamy) macrophages, compared to control cells (Fig. 9a). Notable upregulated genes included complement *C2* and *C3*, *Hla*, *Tnf, Nos2*, while *Il10* was downregulated. Activated upstream regulators, which represent predicted factors that would induce similar signaling pathways, included LPS, IFNγ, TNF, and the infection-associated molecular pattern poly rI:rC-RNA (Fig. 9b). Downregulated upstream regulators included CITED2, an anti-inflammatory negative feedback regulator of NfκB.

Treatment with HPβCD reduced the activity of many of these inflammatory pathways. For example, HPβCD treatment led to downregulation of the macrophage classical activation pathway (Fig. 9c), and reduced the activity of upstream regulators such as LPS, poly rI:rC-RNA and TNF, when compared to stimulated cells (Fig. 9d).

When assessing changes induced by treatment with PZ8059, we saw upregulation of the wound healing pathway, including genes *Col6a3* (collagen), *Il1a*, *1l1b*, *Tnf*, and *Vegfa*. We did not see the reduced activity of inflammatory pathways, such as what was seen following treatment with HPβCD. Instead, treatment with PZ8059 resulted in the upregulation of the IL-17 signaling pathway (Fig. 9e), and activation of inflammatory upstream regulators, including LPS, TNF, NfκB, and several interleukins (Fig. 9f).

When comparing the two CD formulations, PZ8059 elicited greater activation of inflammatory signaling pathways relative to HPβCD. These included the macrophage classical activation pathway, and the IL-17 signaling pathway (Fig. 9g). Furthermore, PZ8059-treated cells showed activation of inflammatory regulators such as LPS, TNF, MyD88 and IL-1β.

## Discussion

Phagocytosis of myelin debris by microglia and macrophages is crucial for repairing demyelinated lesions, but if unregulated, it can exacerbate disease. Our study revealed that myelin phagocytosis begins with an increase in both myelin and free cholesterol staining, followed by a rise in esterified lipids and lipid droplet accumulation. Exposure of macrophages to myelin and inflammatory cytokines induces a foamy, inflammatory phenotype. Notably, both commercially-available and novel CD formulations effectively reduced lipid accumulation within microglia and macrophages, including foamy macrophages *in vitro*. Bulk RNA sequencing revealed that HPβCD reversed several inflammatory pathways that were upregulated in foamy macrophages, while PZ8059 promoted activity of the wound healing pathway.

In mouse macrophages and human microglia, we observed a decline in the expression of MBP and free cholesterol at the 3 d timepoint, a pattern not observed in murine microglia (Fig. 2). This unexpected finding suggests ongoing phagocytosis of myelin debris and persistent association between myelin and murine macrophages, which was not detected in other cell types. If this signal reflected impaired lipid processing, such that the lipids remain MBP + rather than being degraded and esterified, we would expect a smaller increase in the percentage of cells that are BODIPY+, Plin2+ or ORO+, which was not the case. Additionally, if this signal reflected an association between myelin and macrophages without internalization, we would anticipate low levels of free cholesterol, which we did not observe. The basis for this discrepancy between human microglia and mouse macrophages, relative to murine microglia remains unclear.

Our initial use of the Amplex™ Red Cholesterol Assay Kit was insufficient for quantifying cholesterol in cell culture supernatants, due to sequestration of cholesterol by CDs. When we subsequently employed liquid chromatography-mass spectrometry, which was not designed to differentiate free cholesterol from cholesterol-CD complexes, we did not observe differences in supernatant cholesterol levels between untreated and PZ8059-treated macrophages. Besides the technical challenge of possible cholesterol – CD complexes which lowered detection of free cholesterol, several explanations may account for the observed reduction in intracellular content of lipids, without a corresponding increase in extracellular cholesterol. One possibility is that CDs promote the redistribution of cholesterol from lysosomal compartments to cellular membranes, altering intracellular content without increasing cholesterol efflux. Alternatively, CDs may facilitate the conversion of cholesterol into other lipid species, such as oxysterols. Given that cholesterol serves as a precursor for multiple biosynthetic pathways, it is also possible that CD treatment upregulates pathways that promote the utilization of cholesterol for downstream synthesis. Finally, it is possible that CDs did not promote efflux of cholesterol from foam cells in our study. Future experiments are required to investigate the effects of CDs on cholesterol trafficking, metabolism, and lipid accumulation in foamy macrophages.

The impacts of CD treatment on macrophage phenotype were demonstrated through bulk RNA sequencing, where HPβCD led to downregulation of inflammatory pathways and PZ8059 promoted a wound healing pathway (Fig. 9). This wound healing pathway consists of *Col6a3* (collagen) and angiogenesis factor *Vegfa*, as well as pro-inflammatory mediators such as *Il1a*, *Il1b*, and *Tnf*. We also observed increased upregulation of inflammatory upstream regulators such as MyD88 following treatment with PZ8059. Of note, inflammation, including involvement of the MyD88 signaling pathway, is required for some protective functions of macrophages to occur, including phagocytosis, and lack of inflammation can impede remyelination [44,45]. Furthermore, the inflammatory cytokine IL-1β has been shown to be critical for remyelination, as mice lacking this cytokine exhibit impaired remyelination [46]. Thus, one hypothesis is that PZ8059 could ‘activate’ immune cells to carry out beneficial roles.

Several factors may explain the differences in activity observed across the various CD formulations. First, we noted cell toxicity in several formulations, including AC3119 (Fig. 6). Analysis of its chemical structure revealed that AC3119 contains multiple hydrophobic groups, which may facilitate its incorporation into the plasma membrane. Once in the plasma membrane, AC3119 may complex cholesterol and other lipids, disrupting lipid rafts, destabilizing the membrane, and ultimately causing cell death. When comparing HPβCD to the three novel, effective formulations – PZ7061, PZ7086 and PZ8059 – one key difference is that while HPβCD lacks any charges, the novel formulations carry negative charges. These negatively charged novel formulations more closely resemble the commercial sulfobutyl ether β-CD (SEβCD), which has minimal ability to sequester cholesterol in the plasma membrane [47]. Additionally, SEβCD has been shown to interact with glycosphingolipids, another component of myelin debris [48], whereas HPβCD does not. Based on these observations, we hypothesize that our novel formulations could facilitate the clearance of myelin-derived cholesterol and other lipids from microglia and macrophages in MS lesions, without disrupting the plasma membranes of these cells.

Earlier work has measured lipid accumulation in macrophages after myelin exposure, assessing ORO, BODIPY, Plin2 and Filipin after 24 h and ORO, BODIPY, and Filipin after 72 h. [9,[49], [50], [51]]. Prior research generally reported similar trends in BODIPY, ORO and Plin2 expression, with the presence of lipid droplets increasing over time. One exception involves filipin staining, which was previously found to progressively accumulate with time, whereas our findings indicate that filipin levels increased acutely but then decreased by 3 d [9]. One possible reason for this discrepancy is the amount of myelin used. While our study employed a final concentration of 30 μg/ml, the previous study used 100 μg/ml, which may have caused an overload of free cholesterol that could not be properly broken down or processed.

CDs have been widely studied for lipid clearance in disorders such as NPC1 and other neurological diseases [23,52,53]. In the context of myelin-laden macrophages, one previous study demonstrated that methyl-β-cyclodextrin was able to deplete free, but not esterified cholesterol from these cells [9]. As esterified cholesterol is the primary lipid species in foamy macrophages, this suggests a limited therapeutic potential. Moreover, this study did not assess the impact of methyl-β-cyclodextrin on lipid accumulation in foamy macrophages (exposed to both myelin and cytokines), or microglia, a key cell type in MS pathology. Our work expands on previous findings by investigating a more therapeutically relevant CD formulation and additional cell types.

Several limitations of the present study should be considered when interpreting our findings. First, macrophage viability following CD treatment was quantified using the number of DAPI + cells, rather than a viability assay. As a result, this has limited our ability to draw conclusions regarding the effects of CDs on cell viability. Future studies should include assays of cell stress and apoptosis to directly measure how CD treatment impacts macrophage viability.

In addition, our RNA-seq analysis assessing transcriptional changes induced by novel and pre-existing CDs was largely exploratory, which limits mechanistic conclusions. Future studies should focus on validating specific targets of interest, particularly those related to lipid handling and inflammation, to better define how cyclodextrins modulate these pathways in macrophages.

Foamy, lipid-laden macrophages constitute a large proportion of cells within MS lesions, and contribute to disease by promoting an inflammatory microenvironment, and exhibiting reduced phagocytic capacity, which hinders repair [9,11]. Effective remyelination requires functional microglia/macrophage to clear inhibitory myelin debris, and pre-clinical studies in MS models that have pharmacologically enhanced phagocytic activity have shown improved remyelination [1,54]. Our findings demonstrate that CDs can reduce lipid burden in foamy macrophages, and modify the expression of genes associated with inflammation and wound healing. Thus, CDs may have therapeutic potential by altering the inflammatory phenotype of microglia/macrophages and promoting more effective phagocytic function.

In conclusion, foamy macrophages are known to release pro-inflammatory cytokines, and to exhibit reduced phagocytic capacity. It is reasonable to assume that the presence of these macrophages likely perpetuates MS disease and hinders repair processes. Our study highlights the potential of CDs to reduce the accumulation of lipids and modulate foamy macrophage phenotype in the context of MS. Future research will explore the impact of CDs in animal models of MS, investigating its impact on remyelination and repair. This is a necessary step prior to the translation of cyclodextrins into clinical trials of people with MS.

## Author contributions

ECW co-conceived the project, performed the majority of experiments, and wrote the first and final drafts. PZ, AC and CCL provided cyclodextrins and expertise of their chemistry. CB and MTM conducted several tissue culture experiments for this manuscript. CDM co-led the bulk RNA sequencing analyses. VWY co-conceived the project, provided overall supervision, and finalized the manuscript. All authors edited and approved the paper.

## Declaration of competing interest

The authors declare the following financial interests/personal relationships which may be considered as potential competing interests: CCL and PZ declare that they are co-inventors on an awarded patent (US Patent 10, 662, 260) for PZ7061, PZ7084, PZ7086 and PZ7095. They applied for and abandoned the patent application (US Patent App. 15/766,785) for PZ8059, PZ8061, PZ9078 and PZ9080. These are cited as references #28 and #29, respectively, in this text.
