# Supplementary material for: Myelin debris uptake by macrophages and microglia: Resolution of foam cells with a series of novel cyclodextrins
Source: Neurotherapeutics. 2026 Jun 12;23(4):e00943. doi: 10.1016/j.neurot.2026.e00943 (PMC13277444; doi:10.1016/j.neurot.2026.e00943)
Supplement: Multimedia component 1 [file mmc1.pdf]

Supplementary Table 1

Gene	Log2FC	padj	Gene	Log2FC	padj
**Gja4**	9.81	7.97E-21	**Plekhb1**	7.11	9.82E-44
**Cxcl10**	9.71	0	**Pacsin1**	7.01	1.36E-15
**Gm56487**	9.68	5.10E-21	**Klri1**	6.99	7.84E-15
**Cxcl9**	9.44	0	**Lrcol1**	6.98	1.68E-09
**Tgtp1**	9.18	1.02E-159	**Gbp11**	6.95	0
**Upp1**	8.82	0	**Vwa3b**	6.87	3.53E-09
**Serpina3g**	8.71	0	**Gm9385**	6.85	8.80E-08
**Serpina3i**	8.54	5.58E-23	**Trim72**	6.75	1.33E-09
**Gbp5**	8.44	0	**Gbp7**	6.73	0
**Gbp10**	8.41	1.19E-95	**Ptgs2**	6.72	0
**Lrrc14b**	-8.31	5.15E-11	**Tgtp2**	6.68	0
**Chp2**	-8.26	4.29E-14	**Ccl12**	6.61	1.58E-46
**Pdyn**	8.10	7.98E-13	**Mycl**	6.49	3.26E-51
**Dnmt3c**	8.07	9.58E-14	**Klri2**	6.43	1.50E-28
**Cxcl11**	8.05	3.07E-92	**Ccl19**	6.43	3.29E-08
**Serpina3f**	7.98	0	**Insrr**	6.29	8.95E-08
**Klrk1**	7.81	3.58E-107	**Dnah5**	6.29	1.93E-08
**Iigp1**	7.77	0	**Pappa**	-6.24	4.24E-07
**Gm43802**	7.69	8.21E-144	**Apol10b**	6.22	4.31E-134
**Nos2**	7.53	0	**BC023105**	6.18	1.75E-37
**Gm43302**	7.50	6.40E-271	**Hcar2**	6.18	0
**Gbp4**	7.44	0	**Ubd**	6.11	0
**Gm47803**	7.40	3.04E-09	**Gm4841**	6.01	0
**Selp**	7.25	2.60E-13	**Gm9795**	-6.01	2.09E-03
**Socs1**	7.13	0	**Mobp**	5.98	9.70E-256

Supplementary Table 2

Gene	Log2FC	padj	Gene	Log2FC	padj
**Gm2436**	5.46	3.21E-02	**Epha4**	-1.68	1.95E-07
**Mex3b**	4.97	1.67E-166	**Gm28041**	-1.67	2.66E-02
**Kctd15**	-3.71	1.73E-02	**Cyp2j13**	1.67	5.29E-03
**Six1**	-2.79	2.54E-06	**Rad51b**	1.67	2.66E-02
**Ccdc80**	-2.76	9.96E-03	**Map2k6**	1.67	1.13E-03
**Lad1**	-2.59	9.33E-06	**Cxcr3**	1.67	2.14E-17
**Htr7**	-2.57	1.26E-04	**Fzd1**	-1.66	8.39E-81
**Il10**	2.52	5.34E-08	**Ablim2**	-1.66	3.81E-11
**Gm27029**	2.48	2.89E-04	**Grap2**	1.66	8.89E-03
**Cxcl12**	2.30	5.39E-17	**Adora2a**	-1.63	2.34E-10
**Zbtb16**	2.30	1.20E-02	**Ptpn3**	-1.59	1.29E-02
**Dlec1**	2.21	1.54E-03	**Rhobtb1**	1.59	1.86E-12
**Rasal1**	-2.13	2.11E-02	**Lyrm7**	1.57	7.27E-03
**Bhlhb9**	2.10	2.20E-03	**Arrdc3**	1.57	4.97E-24
**Atp6-ps**	-2.02	2.32E-02	**Tmod1**	1.56	1.01E-05
**Vegfa**	1.98	3.07E-210	**Galnt9**	1.56	7.49E-03
**Mycl**	-1.90	9.61E-14	**Ocstamp**	1.55	8.38E-23
**Slamf1**	-1.85	2.72E-09	**6430550D23Rik**	1.55	4.99E-03
**Gm12895**	-1.83	3.96E-02	**Rtn4rl1**	1.55	3.28E-02
**Gm12896**	-1.83	3.96E-02	**Dnah2**	1.53	4.39E-02
**Syt7**	-1.81	1.35E-07	**Spint2**	-1.53	7.22E-08
**Nhsl2**	1.78	1.80E-03	**Aspm**	1.53	1.28E-04
**Gm2007**	-1.71	4.17E-02	**Ddit4l**	-1.53	3.45E-02
**Gm28437**	-1.71	1.24E-02	**Adora2b**	-1.52	1.44E-12
**Ntrk3**	1.70	4.81E-02	**Cd247**	-1.50	5.13E-07

Supplementary Table 3

Gene	Log2FC	padj	Gene	Log2FC	padj
**Gm27029**	2.55	1.04E-03	**Fbln2**	0.89	1.78E-03
**Tmem26**	1.91	2.01E-06	**Grhpr**	0.88	9.40E-06
**Col6a3**	1.78	3.43E-04	**Poc1b**	-0.87	6.92E-06
**Nectin1**	-1.74	1.01E-05	**Tlr11**	-0.87	7.85E-04
**Dhcr24**	-1.74	1.89E-11	**Ggnbp1**	-0.87	1.63E-03
**Cd59b**	-1.71	4.96E-02	**Selp**	-0.86	3.46E-02
**Srl**	-1.57	1.10E-02	**Ipcef1**	-0.86	2.91E-02
**Tfrc**	1.48	3.25E-05	**Vmn2r124**	-0.85	5.37E-03
**Card14**	-1.43	3.25E-03	**Abcd2**	0.82	2.98E-03
**Rcor2**	1.39	1.64E-02	**Nlrp3**	0.82	5.04E-29
**Mgarp**	1.30	1.08E-03	**Fpr1**	0.81	1.35E-02
**Il1a**	1.13	3.96E-08	**Egln3**	0.80	9.51E-05
**Il1b**	1.12	1.18E-03	**Etl4**	-0.79	5.79E-06
**Magi2**	-1.11	3.46E-03	**Plod2**	0.79	7.93E-03
**Ccdc15**	-1.02	3.66E-02	**Mthfd1l**	0.79	9.16E-16
**Aldh1l2**	1.00	2.21E-02	**Cyp51**	-0.79	1.09E-06
**Mex3b**	0.99	3.61E-05	**Ikzf2**	-0.78	4.43E-02
**Cxcl2**	0.94	1.59E-04	**Nfkbiz**	0.77	1.97E-27
**Chtf18**	0.94	6.03E-03	**Cd300e**	0.77	1.42E-04
**Zdhhc23**	-0.93	1.25E-03	**Mzf1**	-0.77	3.84E-02
**Nanos1**	-0.92	4.57E-02	**Vegfa**	0.75	3.53E-29
**Ldlr**	-0.92	2.60E-23	**Ptx3**	0.73	4.94E-02
**Idi1**	-0.90	1.49E-07	**Me3**	-0.73	4.94E-02
**Prelid2**	0.90	2.01E-06	**Ptgs1**	-0.72	1.07E-04
**Cep97**	0.89	3.26E-02	**Slc40a1**	-0.72	4.60E-18

Supplementary Table 4

Gene	Log2FC	padj	Gene	Log2FC	padj
**Gm50388**	-7.10	2.83E-04	**Adora2a**	2.32	7.79E-21
**Gm12191**	-7.07	2.18E-03	**Slamf1**	2.30	3.14E-14
**Gm15710**	-6.53	5.59E-03	**Cxcr3**	-2.24	6.00E-25
**Gm2423**	-6.20	1.62E-02	**Celsr2**	-2.22	2.73E-02
**Gm10780**	6.19	1.06E-03	**Fpr1**	2.17	1.17E-18
**Gm6565**	6.18	4.63E-02	**Il12b**	2.13	2.25E-03
**Mex3b**	-3.98	1.13E-126	**Haao**	-2.04	7.40E-03
**Tafa3**	-3.87	1.30E-02	**Cxcl12**	-1.95	6.39E-13
**Six1**	3.63	1.67E-10	**Slco2b1**	-1.95	3.03E-02
**Slc16a12**	-3.10	1.96E-02	**Ptgs1**	-1.95	2.68E-39
**Lad1**	2.98	1.77E-07	**Adora2b**	1.91	6.78E-20
**Gm15421**	-2.95	4.17E-02	**Ablim2**	1.91	1.22E-14
**Rtn4rl1**	-2.82	3.44E-03	**Atp6-ps**	1.89	3.54E-02
**Acsm3**	-2.78	1.83E-02	**Adcy4**	-1.89	1.50E-03
**Nav3**	-2.67	2.05E-02	**Syt17**	1.88	3.57E-02
**Il10**	-2.65	2.56E-08	**Gm42776**	-1.84	2.33E-02
**Sh3rf3**	-2.65	8.30E-03	**D630023F18Rik**	-1.84	4.87E-02
**Il1b**	2.58	3.42E-17	**Ipcef1**	-1.84	9.62E-11
**Rnase10**	2.56	3.47E-03	**1810049J17Rik**	1.82	2.01E-06
**Mycl**	2.47	3.38E-23	**Otos**	-1.81	1.42E-02
**Mmp10**	2.46	3.07E-04	**Tlr11**	-1.80	2.07E-18
**Cst6**	2.46	3.46E-02	**Gem**	1.80	3.14E-03
**Htr7**	2.44	3.25E-04	**Nat8l**	-1.79	1.24E-03
**Kbtbd11**	-2.41	3.58E-03	**Il6**	1.78	7.87E-09
**Grap2**	-2.38	2.83E-04	**Cxcl2**	1.76	1.47E-16
