# Supplementary material for: Myelin debris uptake by macrophages and microglia: Resolution of foam cells with a series of novel cyclodextrins
Source: Neurotherapeutics. 2026 Jun 12;23(4):e00943. doi: 10.1016/j.neurot.2026.e00943 (PMC13277444; doi:10.1016/j.neurot.2026.e00943)

Supplementary Fig. 1

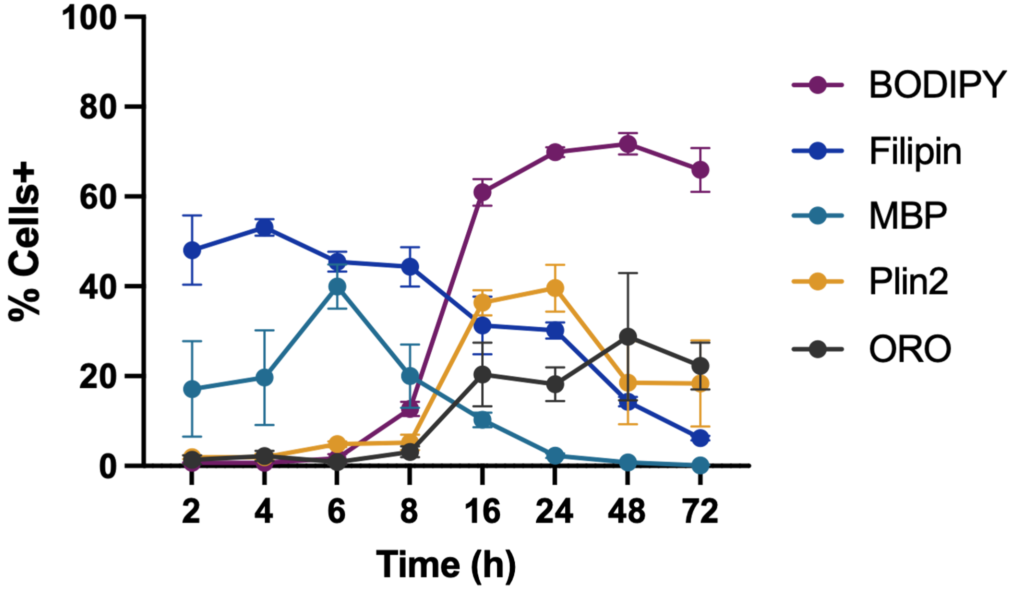

Supplementary Fig. 2

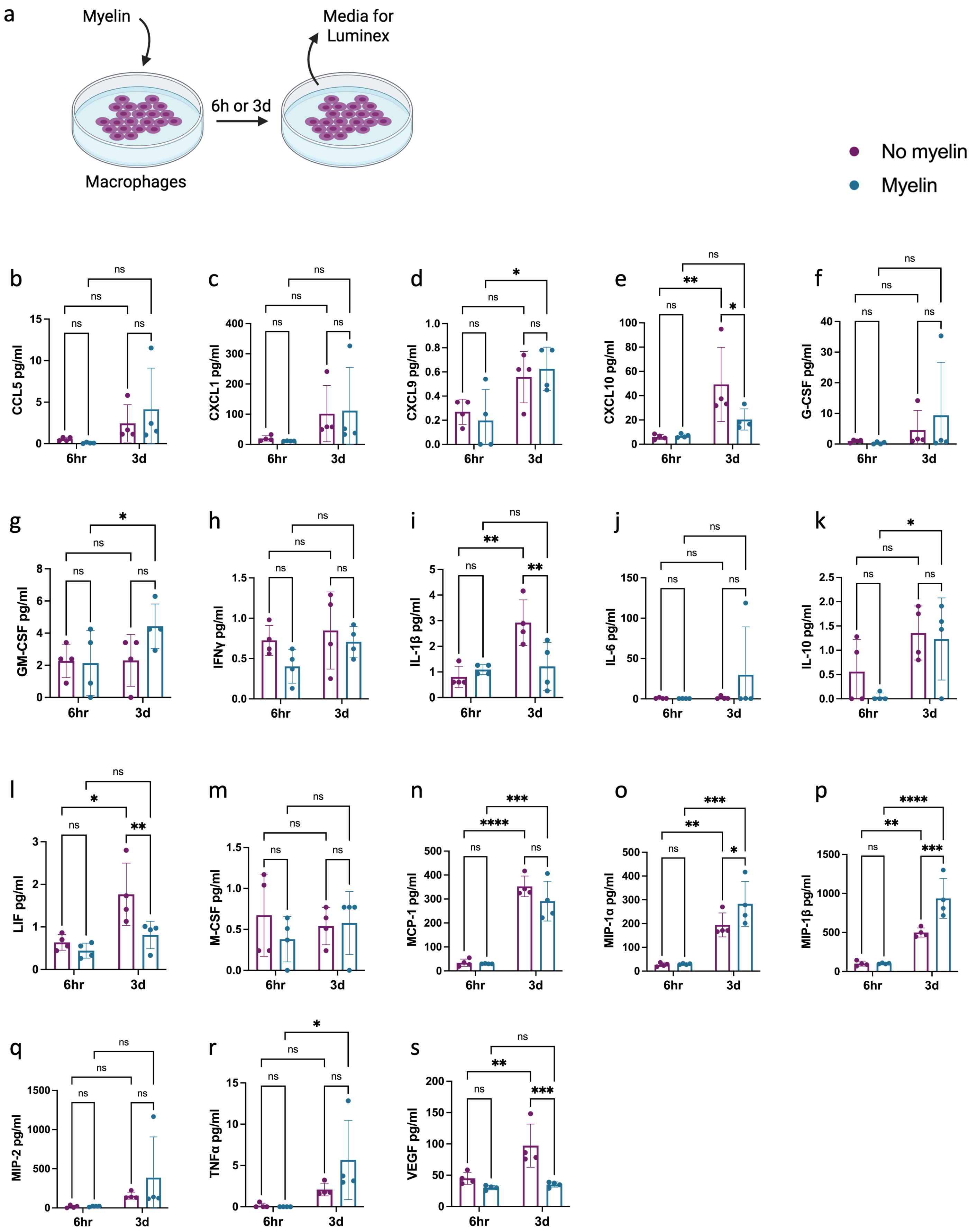

Supplementary Fig. 3

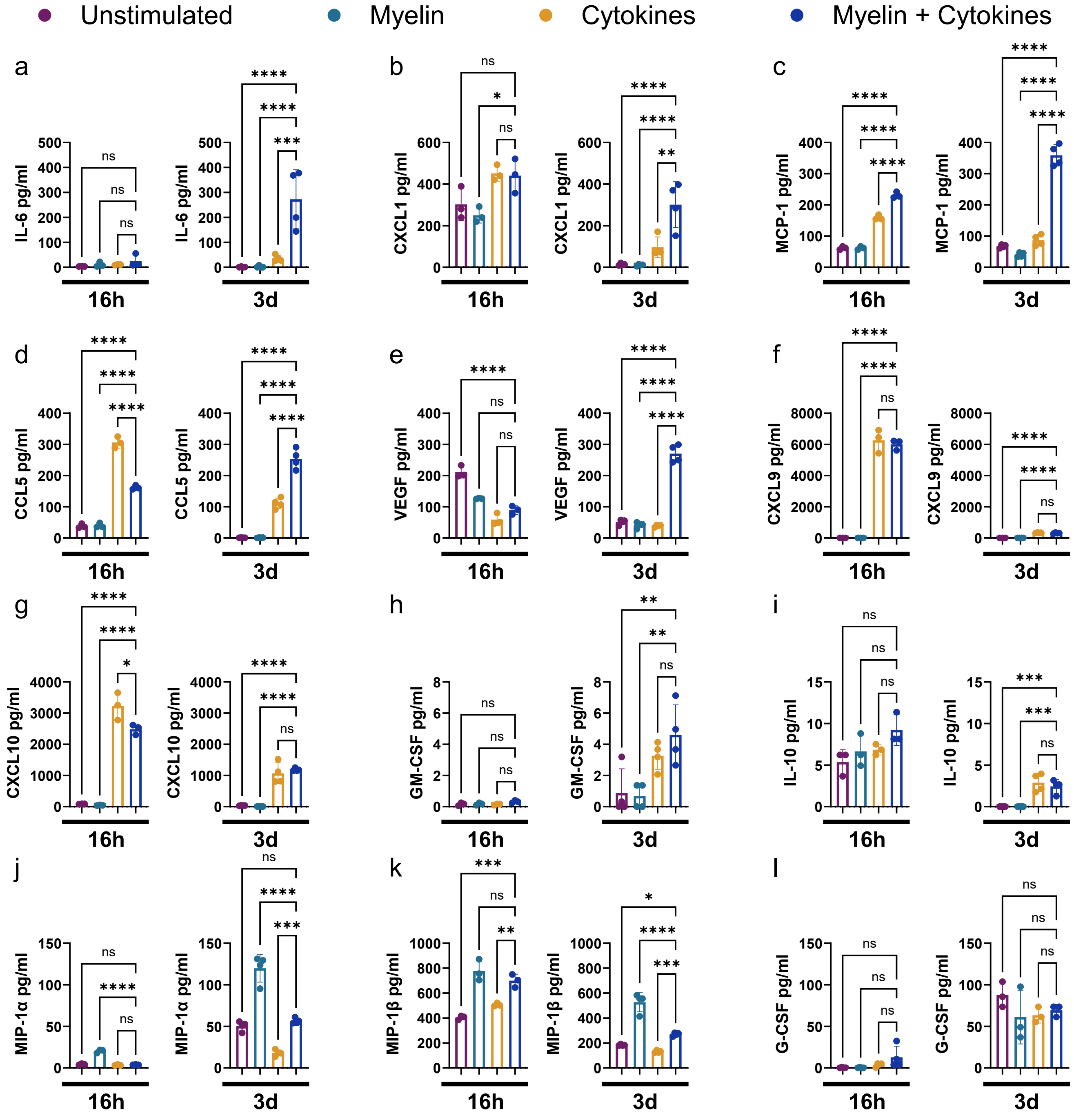

Supplementary Fig. 4

a

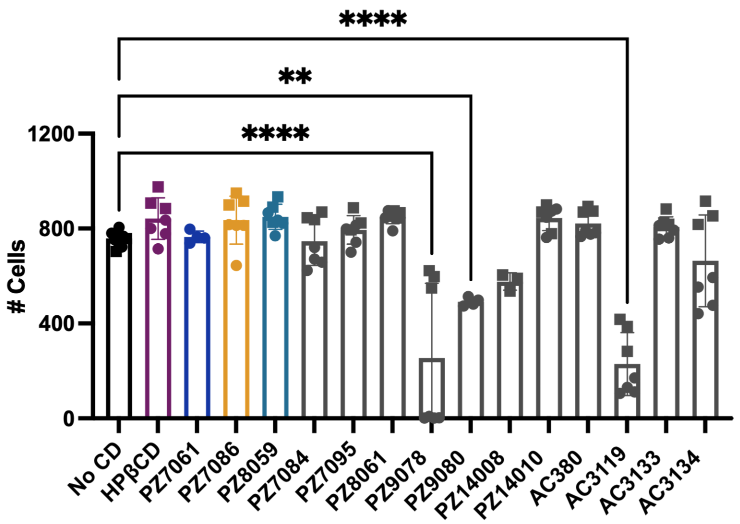

b

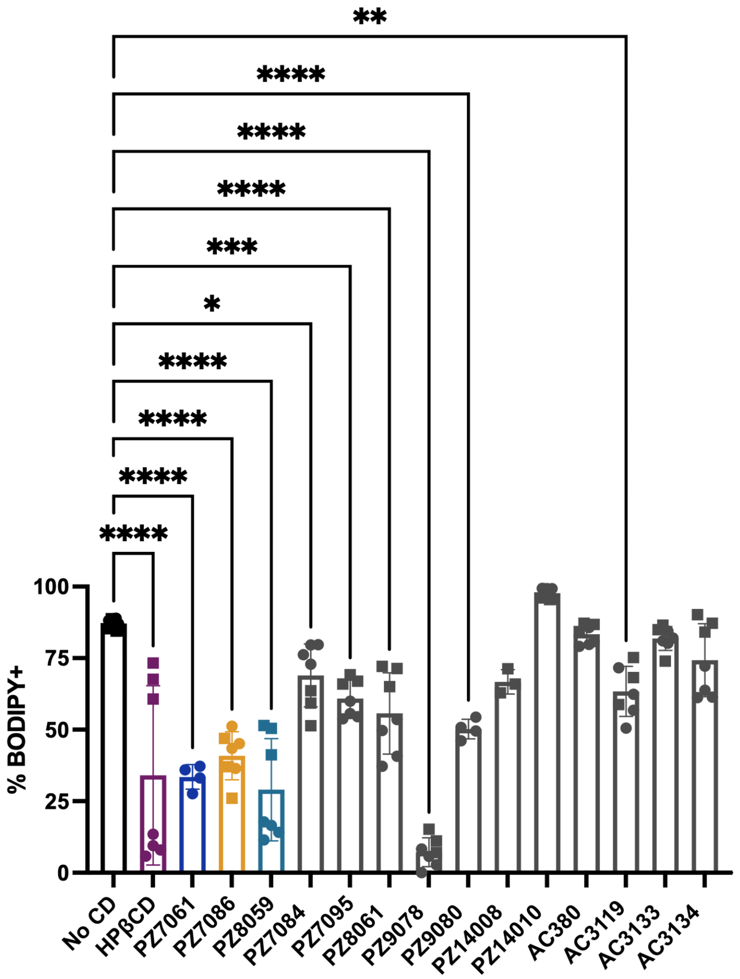

Supplementary Fig. 5

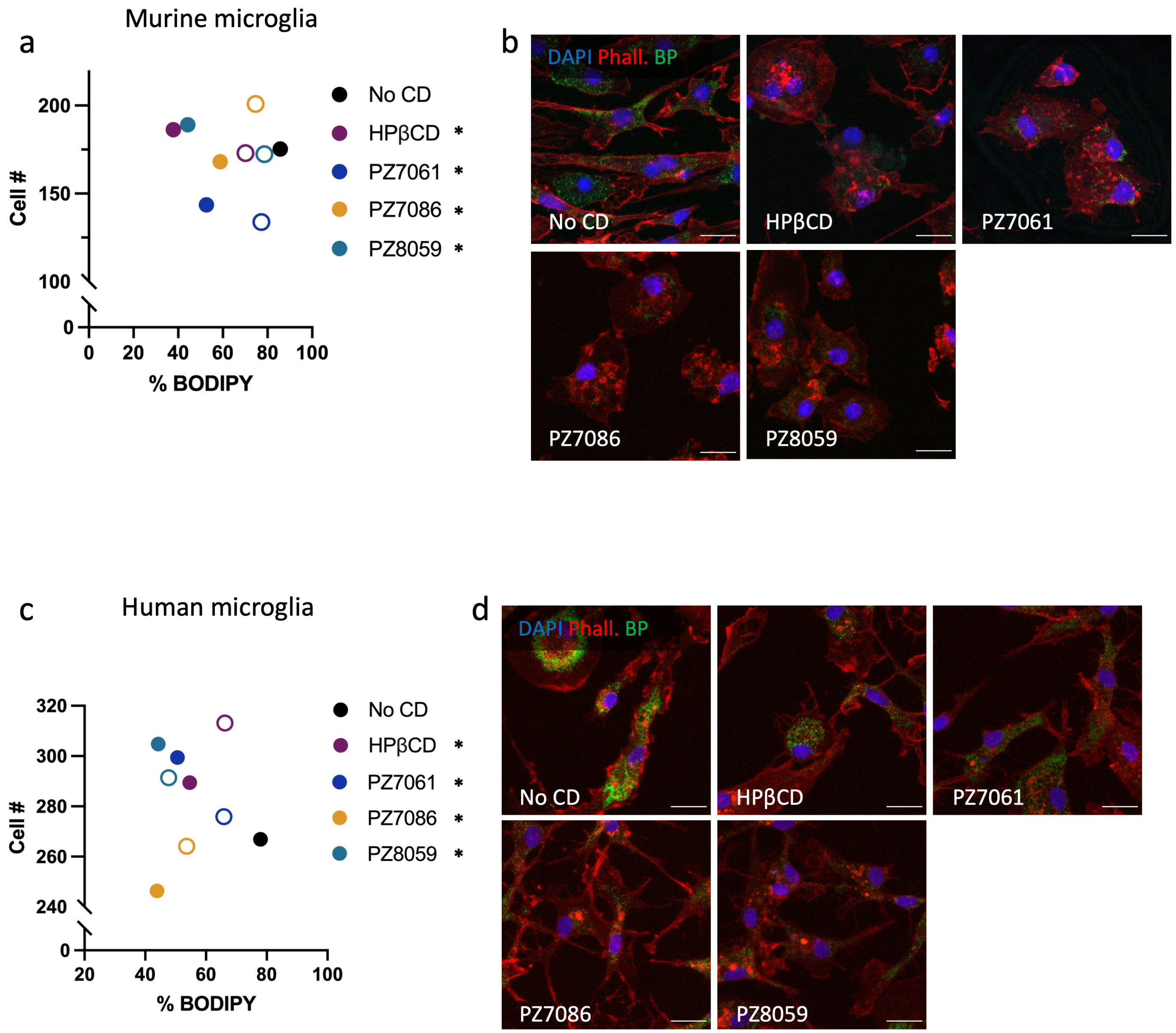

Supplementary Fig. 6

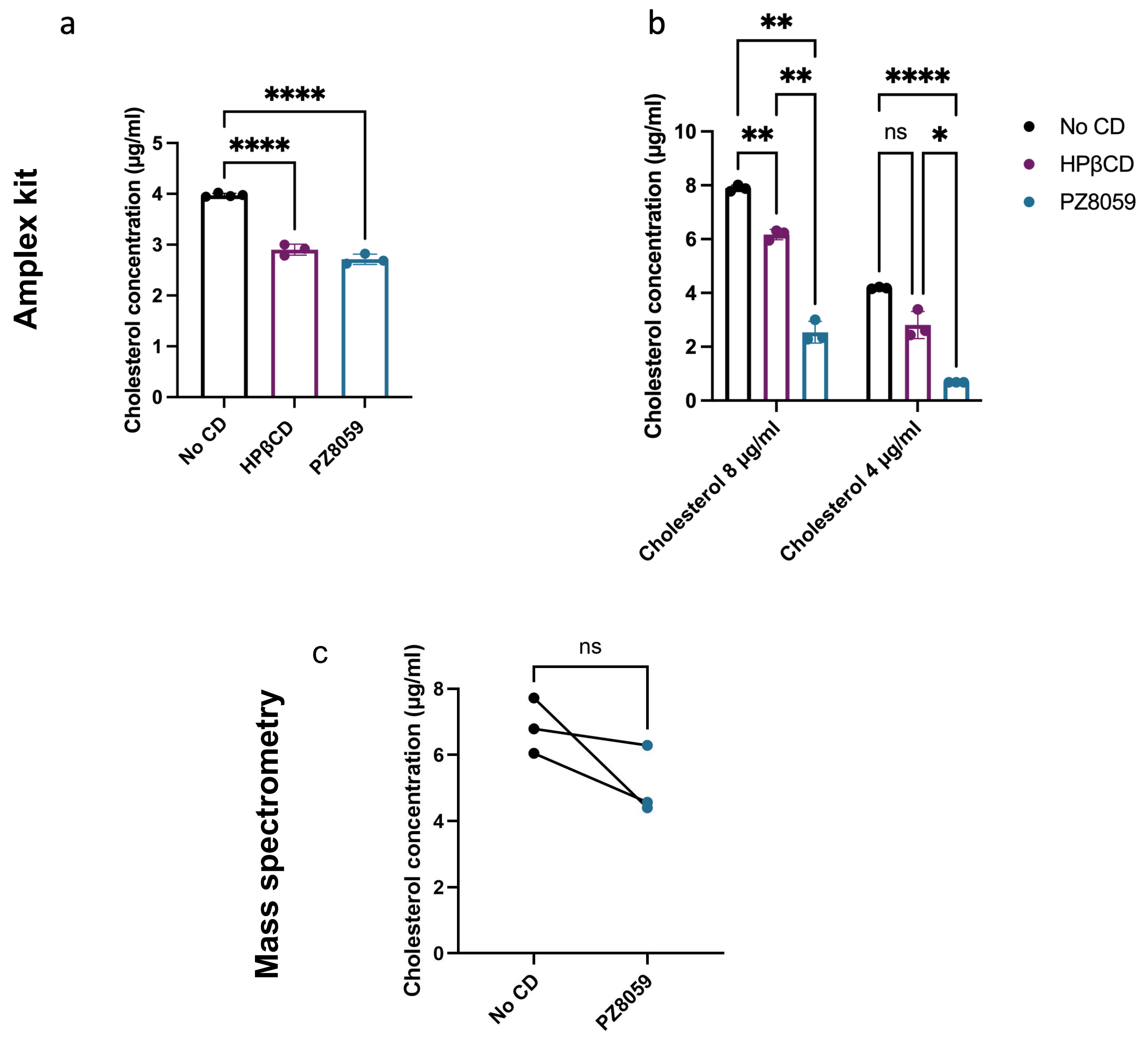

Supplementary Fig. 7

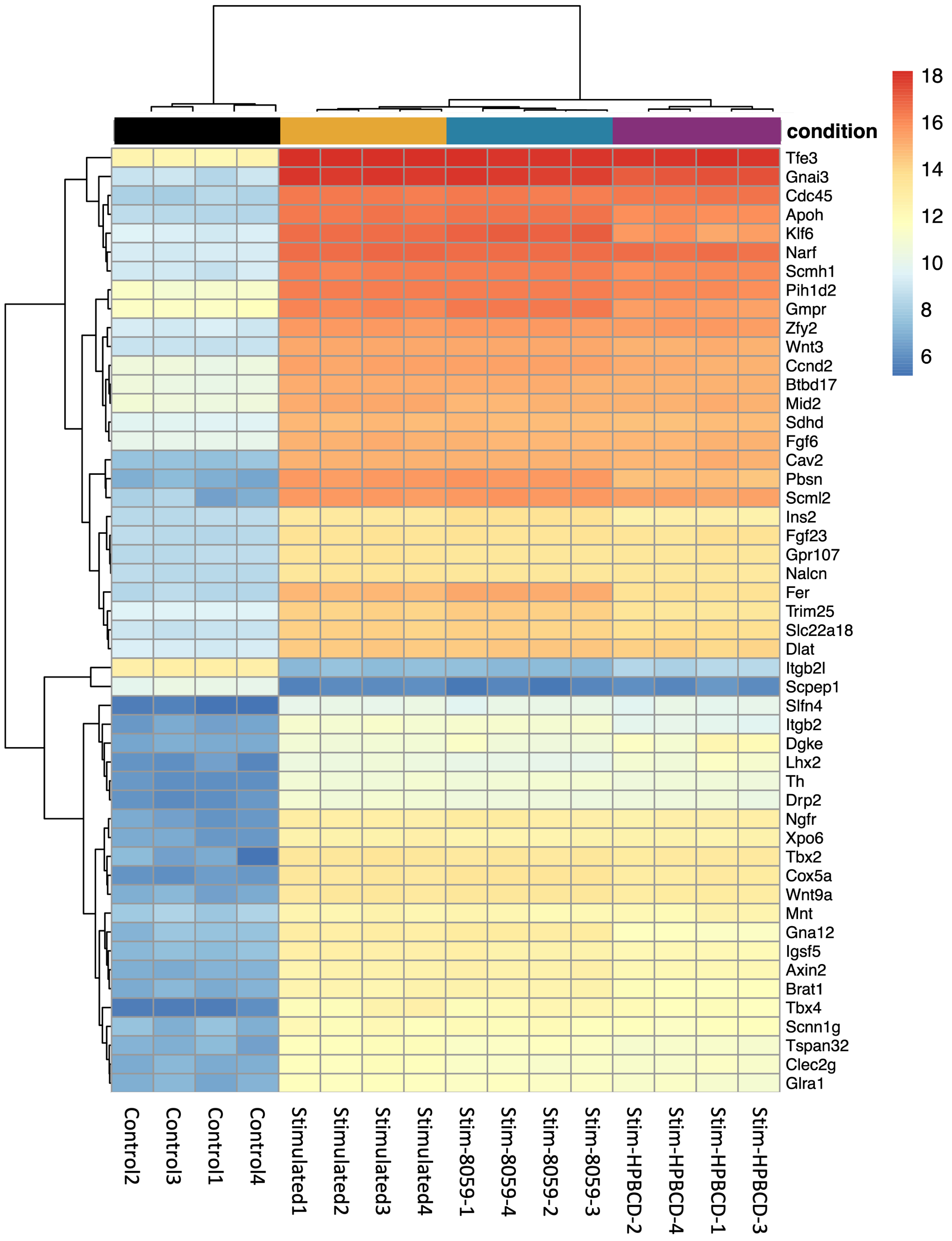

Supplement: Multimedia component 2 [file mmc2.pdf]
