# Supplementary material for: Myelin debris uptake by macrophages and microglia: Resolution of foam cells with a series of novel cyclodextrins
Source: Neurotherapeutics. 2026 Jun 12;23(4):e00943. doi: 10.1016/j.neurot.2026.e00943 (PMC13277444; doi:10.1016/j.neurot.2026.e00943)
Supplement: Multimedia component 3 [file mmc3.docx]

**Supplementary information**

**Supplementary Fig. 1 Characterizing myelin debris uptake across time.** Time course showing the levels of lipid markers from 2 to 72 h after myelin application. Each datapoint is an average of 3-4 technical replicates. Experimental was not repeated. Mean +/- SD.

**Supplementary Fig. 2 Detection of cytokines and chemokines in supernatant of myelin-exposed macrophages. a)** Schematic of experimental design. **b-s)** Quantification of the following cytokines and chemokines in the supernatant of macrophages, with or without myelin, at acute and chronic time points: CCL5 **(b)**, CXCL1 **(c)**, CXCL9 **(d)**, CXCL10 **(e)**, G-CSF **(f)**, GM-CSF **(g)**, IFNγ **(h)**, IL-1β **(i)**, IL-6 **(j)**, IL-10 **(k)**, LIF **(l)**, M-CSF **(m)**, MCP-1 **(n)**, MIP-1α **(o)**, MIP-1β **(p)**, MIP-2 **(q)**, TNFα **(r)**, VEGF **(S)**. Mean +/- SD. *p<0.05 **p<0.01 ***p<0.001 ****p<0.0001. Two-way ANOVA with Fisher’s LSD post-hoc test. Schematic created with BioRender.

**Supplementary Fig. 3 Detection of additional cytokines and chemokines in macrophage cell culture supernatant following exposure to myelin and/or cytokines. a-m**) Measurement of the following cytokines and chemokines in the supernatant of macrophages exposed to myelin and/or cytokines at post-acute (16 h) and chronic (3 d) time points: IL-6 (a), CXCL1 (b), MCP-1 (c), CCL5 (d), VEGF (e), CXCL9 (f), CXCL10 (g), GM-CSF (h), IL-10 (i), MIP-1α (j), MIP-1β (k), G-CSF (l). Mean +/- SD. *p<0.05 **p<0.01 ***p<0.001 ****p<0.0001. One-way ANOVA with Tukey’s post-hoc test.

**Supplementary Fig. 4 Quantitative assessment of cyclodextrin effects on cell number and BODIPY staining.** Data was generated from the experiments shown in Fig. 6 scatter plot. Quantifications of the number of DAPI+ cells **(a)** or percentage of DAPI+ cells that were BODIPY+ **(b).** All data are from cells exposed to cyclodextrins at a concentration of 5mM. Circles represent data from one experiment, and squares represent data from a second experiment. Due to the availability of novel cyclodextrin formulations, some formulations were tested in only one experiment. Mean +/- SD. *p<0.05 **p<0.01 ***p<0.001 ****p<0.0001. One-way ANOVA with Tukey’s post-hoc test.

**Supplementary Fig. 5 Cyclodextrins reduce intracellular lipid accumulation in murine and human microglia a, c)** Scatter plot representing the number of live murine **(a)** or human **(c)** microglia versus the percentage of cells positive for lipid droplet marker BODIPY. Each dot represents one tested formulation, with closed circles representing 5mM and open circles representing 1mM concentration. **b, d)** Representative images of untreated or cyclodextrin-treated murine **(b)** or human **(d)** microglia stained with BODIPY. Scale bar indicates 20 µm. Images were taken from wells treated with 5mM of cyclodextrins.

**Supplementary Fig. 6 Measurement of cholesterol concentration in cell culture supernatant. a)** Quantification by Amplex Red of cholesterol in cell culture supernatant of untreated foamy macrophages, or foamy macrophages treated with HPBCD or PZ8059. **b)** Quantification of cholesterol by by Amplex Red in standard curve wells, with or without the addition of cyclodextrins. For both experiments, CDs were added at a concentration of 5mM. Mean +/- SD. *p<0.05 **p<0.01 ****p<0.0001. N=3 independent experiments; each experiment included 3-4 replicates for work on cholesterol concentration in CD-exposed media (Panel a). One-way ANOVA with Tukey’s post-hoc test. **c)** Quantitation of cholesterol in control or PZ8059-treated macrophages by mass spectrometry, in 3 cultures from 2 experiments. The joined lines represent macrophages in control condition or when treated with CD. ns: not significant (paired t-test).

**Supplementary Fig. 7 Heatmap of differentially expressed genes.** Plot was generated with hierarchical clustering on rlog-transformed data, and displays differences in gene expression across the top 50 most variable genes.

**Supplementary Table 1 Top 50 Differentially expressed genes in stimulated macrophages versus controls**

**Supplementary Table 2** **Top 50 differentially expressed genes in HPβCD-treated macrophages versus stimulated**

**Supplementary Table 3 Top 50 differentially expressed genes in PZ8059-treated macrophages versus stimulated**

**Supplementary Table 4 Top 50 differentially expressed genes in PZ8059-treated versus HPβCD-treated macrophages.**
